# Identification of functional genes in liver fibrosis based on bioinformatics analysis of a lncRNA-mediated ceRNA network

**DOI:** 10.1186/s12920-024-01813-x

**Published:** 2024-02-20

**Authors:** Feng Zhang, Siya Pei, Meifang Xiao

**Affiliations:** 1 Department of Cardiovascular Medicine, Xiangya Hospital, Central South University, Hunan Changsha, 410008 People’s Republic of China; 2 National Clinical Research Center for Geriatric Disorders, Xiangya Hospital, Central South University, Hunan Changsha, 410008 People’s Republic of China; 3 Department of Infection Diseases, Xiangya Hospital, Central South University, Hunan Changsha, 410008 People’s Republic of China; 4 Department of Health Management Center, Xiangya Hospital, Central South University, Hunan Changsha, 410008 People’s Republic of China

**Keywords:** Liver fibrosis, Bioinformatics analysis, lncRNA, ceRNA, Perl

## Abstract

**Background:**

Liver fibrosis is a major global healths problem; nevertheless, its molecular mechanism are not completely clear. This study aimed to build a lncRNA-miRNA-mRNA network, identify potentially related lncRNAs, and explore the pathogenesis of liver fibrosis.

**Materials and methods:**

We used the Gene Expression Omnibus databases and bioinformatics analysis to identify differentially expressed genes (DEGs) between liver fibrosis and normal tissues. The ceRNA network was constructed according to the interactions between DElncRNA, miRNA, and DEmRNA. Then, these DEGs were identified using functional enrichment analysis, and a protein–protein interaction (PPI) network was established. The critical lncRNAs were verified using the quantitative real-time polymerase chain reaction (qRT-PCR).

**Results:**

The ceRNA network was composed of three lncRNAs, five miRNAs, and 93 mRNAs. Gene Ontology functional enrichment analysis revealed significant enhancement in cell components, molecular function, and biological process. Kyoto Encyclopedia of Genes and Genomes pathway analysis revealed pathways associated with transcriptional misregulation in cancer, including the Rap1 signaling pathway, proteoglycans in cancer, mineral absorption, HTLV-l infection, and central carbon metabolism in cancer. According to the PPI network and the GSE84044 database, seven hub genes associated with liver fibrosis were identified. In addition, qRT-PCR revealed that lncRNA AC100861 (lncRNA TNFRSF10A-DT) was explicitly decreased in liver fibrosis tissues and activated hepatic stellate cells.

**Conclusions:**

In summary, this study preliminarily found that lncRNA TNFRSF10A-DT may be a biomarker for the diagnosis and outcome of liver fibrosis. We uncovered a novel lncRNA-mediated ceRNA regulatory mechanism in the pathogenesis of liver fibrosis.

**Supplementary Information:**

The online version contains supplementary material available at 10.1186/s12920-024-01813-x.

## Introduction

Liver fibrosis is a common event that triggers wound-healing in the context of chronic injury. Fibrosis may progress to cirrhosis and primary liver cancer [1]. Studies reported that liver fibrosis is associated with the deposition of extracellular matrix (ECM) proteins [2]. The activation of hepatic stellate cells (HSCs) is thought to be the primary factor causing liver fibrosis [3–6]. Liver fibrosis can be detected early and reversed by treatments [7]. In the past few decades, investigators have focused substantial attention on the molecular mechanisms of liver fibrosis [8, 9]. Nevertheless, precise pathogenesis requires further exploration [10]. Definitive treatment of liver fibrosis requires identifying novel therapeutic strategies that include novel biomarkers and targets.

Long non-coding RNAs (lncRNAs) are regulated by several mechanisms, including chromatin regulation, epigenetic modification promoter activity regulation, and post-transcriptional mechanisms [11–14]. Studies indicated that lncRNA participates in the formation or inhibition of liver fibrosis [15–17]. LncRNAs regulate gene expression in acis or trans manner [18, 19]. Exploring the mechanisms of interactions between lncRNA and miRNA can generate novel strategies for liver fibrosis treatment [20].

The number of lncRNA families is vast, and it remains unknown whether there are other lncRNAs involved in liver fibrosis. Therefore, in this study, the expression of lncRNAs and mRNAs in liver fibrosis were identified using the Gene Expression Omnibus (GEO) database, and a co-expression network was constructed to elucidate the regulation and targets of lncRNAs. Then, we used Gene Ontology (GO) terminology and Kyoto Encyclopedia of Genes and Genomes (KEGG) pathway analysis to identify the functions of the lncRNAs associated with liver fibrosis. Finally, we used quantitative real-time polymerase chain reaction (qRT-PCR) to confirm the results.

## Material and methods

### Data collection

This study intended to preliminarily explore the regulatory mechanisms of ceRNA during liver fibrosis progression by analyzing the gene expression differences and co-expression analysis between the healthy group and a liver fibrosis group. GEO, a public international and functional genomics data repository, is used for high-throughput microarray and next-generation sequences [21]. Therefore, GSE12392 and GSE84044 were mined for bioinformatics analysis by searching the keywords "liver fibrosis" and "Homo sapiens" in the GEO database. In the dataset, lncRNA and mRNA expression in six healthy individuals and six patients were measured using the Agilent MicroArray V4 platform.

### DEGs analysis

The DEGs were located using edgeR [22] in the R Bioconductor package. The expression data in GSE12392 were analyzed using the LIMMA package of the R language, and the probe ID was converted into GeneSymbol according to the CORRESPONDING GPL file of the probe. The mRNA or lncRNA were selected from the probe for differential expression and gene co-expression network analysis. The criteria for selecting DEGs were *P* < 0.05 and log2 Fold-Change less than -1 or greater than 1.

### WGCNA analysis

Gene co-expression network analysis was used to analyze gene expression in the two groups at the mRNA and lncRNA levels in combination with basic clinical information of the samples (i.e., age, gender, and whether liver fibrosis occurred) aiming to explore gene co-expression modules related to disease occurrence.

### LncRNAs target gene prediction

The results of co-expression analysis and differential expression analysis were combined to obtain common lncRNAs, that is, to identify DEGs and co-expressed genes associated with clinicopathological features. StarBase and bioinformatics were used to predict the target genes of lncRNAs.

### MiRNA target gene prediction

StarBase and bioinformatics methods were used to predict the target genes of human miRNAs, and the default parameters of the official database were adopted.

### Establishment of the ceRNA regulatory network

An lncRNA-miRNA-mRNA network was constructed according to ceRNA theory [23]. Based on the prediction results of these target genes, a ceRNA regulatory network of lncRNA-miRNA-mRNA was constructed by combining mRNA co-expression network analysis (i.e., genes co-expressed between mRNA level and liver fibrosis) and mRNA differential expression analysis (i.e., genes with differential expression). We used Cytoscape 3.7.2 [24] to visualize the lncRNA-miRNA-mRNA network, and the CytoHubba plugin in Cytoscape 3.7.2 [25] was used to calculate all node degrees.

### GO and pathway enrichment analysis

GO analysis [26] included biological process, cellular component, and molecular function, generated using the bioinformatics tool DAVID [27] (*P* < 0.05). This analysis was used to examine the unique biological significance of the high-throughput transcriptome. KEGG enrichment analysis was used to predict the biological pathways [28]. The results were visualized using the R language.

### Analysis of the PPI network

The PPI information for the common DEG network was evaluated using the search tool STRING [29]. Any potential correlations between these DEGs were analyzed using Cytoscape [30]. The PPI network modules were recognized using the Cytoscape plugin MCODE, and only those were presented based on a node degree ≥ 3.

### Hub gene selection

We analyzed the PPI network using the Cyto-Hubba plugin of Cytoscape 3.7.2 and selected candidate hub genes with top node degrees. Subsequently, hub genes associated with liver fibrosis were identified using GSE84044.

### Human samples

From the Xiangya Hospital of Central South University, ten healthy subjects and 16 patients with liver fibrosis were recruited. Informed consent was obtained, and ethical approval was obtained from the Ethics Committee of Xiangya Hospital. Human liver tissues were immediately frozen in liquid nitrogen and stored at − 80 °C.

### Cell culture experiments

LX-2 cells were obtained from our laboratory and cultured in DMEM containing 1% fetal bovine serum and 1% penicillin/streptomycin. LX-2 cells were passaged using trypsin. LX-2 cells were stimulated by TGF-β1 (0, 5, 10, 20, 40 ng/ml; Sigma, Cat No. SAB4502958) for 48 h. All cells were stored in an incubator at 37 °C in 5% CO_2_.

### QRT-PCR

Total RNA was isolated from liver fibrosis human samples and LX-2 cell lines using TRIzol reagent (Invitrogen, CA, USA). The cDNAs were synthesized using a commercial kit (Bio-Rad, Hercules, CA). Gene expression was measured using the ABI 7900HT Fast Real-Time PCR System. Relative mRNA expression levels were calculated using the 2^−ΔΔCT^ method. The following primer sequences were used: lncRNA PCBP1-AS1, forward, 5'-ACTACTCAGTCAATTGCTCCA-3', reverse, 5'-ATTTCCTTACTGACCTGCAT-3'; lncRNA AC100861, forward, 5'-GCAC ATGACACGGGATGAGA-3', reverse, 5'-GGCTTTCGGGAGGCTGATT A-3'; lncRNA TEX41, forward, 5'-TGGCCAAGAGACAACACCAA-3', reverse, 5'-GGCAGAGTGAGTCCAAAGG-3'; GAPDH, forward, 5'-TGGAAATCCCATCACCATCT-3', reverse, 5'-TGGACTCCACGACGTACTCA-3'.

### Statistical analysis

Data obtained from three independent experiments were expressed as mean ± standard deviation. The *t*-test was performed to compare differences between two groups, and one-way analysis of variance was used for multi-group comparisons. Differences with *p* < 0.05 were considered statistically significant in multiple testing.

## Results

### Identification of differentially expressed genes (DEGs)

**Fig. 1:**
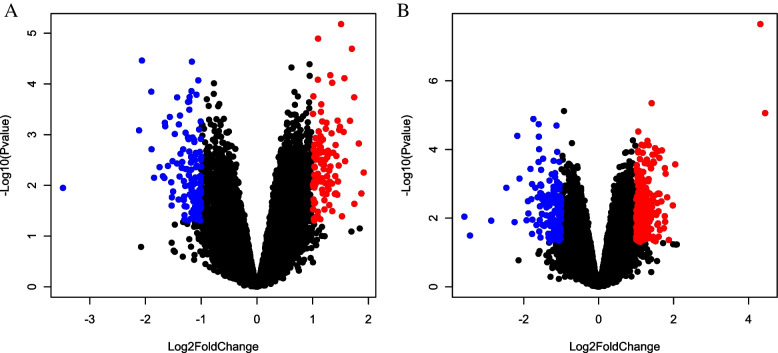
Volcano map demonstrating the differential expression analysis of lncRNAs (**A**) and mRNAs (**B**). Red and blue dots were significantly high and low expression in the disease group, respectively

**Fig. 2:**
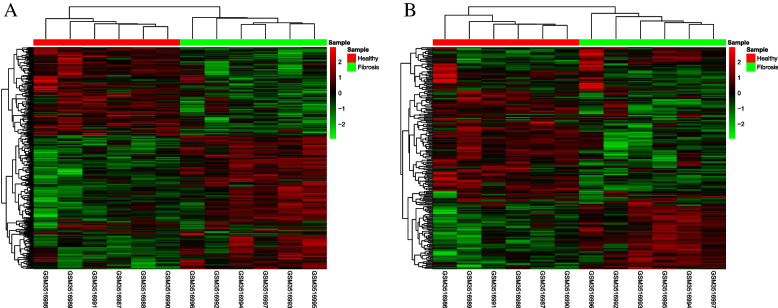
Heat map illustrating differential expression analysis of lncRNAs (**A**) and mRNAs (**B**)

The differential expressions of mRNA and lncRNA in the GSE123932 data set were analyzed using the R language's LIMMA package. The standard of differential gene screening was *P* < 0.05, log2 fold change > 1 or < -1. There were 210 cases with significantly differentially expressed lncRNAs (Figs. 1A and 2A); there were 333 cases of significantly differentially expressed mRNAs (Figs. 1B and 2B).

### Weighted gene co-expression network analysis(WGNA)

**Fig. 3:**
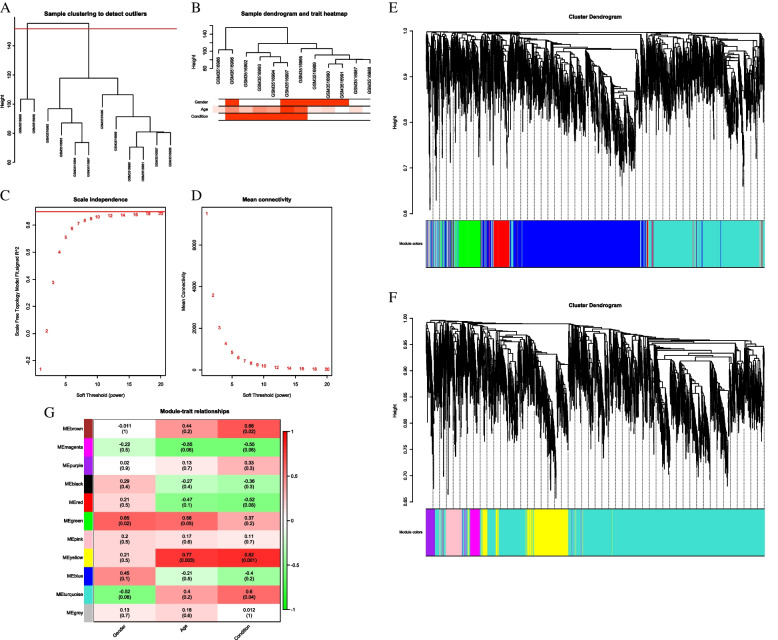
Sample cluster analysis (**A** and **B**); Calculation and selection of soft threshold (**C** and **D**); Cluster analysis of modules (**E** and **F**); Correlation analysis between modules and traits (**G**).

mRNA. None of the 12 samples showed abnormal gene expression (Fig. 3A). Cluster analysis of gene expression profiles was performed by combining clinical information of samples (Fig. 3B). The experimental results converged when the Soft threshold (SFT) value was 8 (Fig. 3C, D). Based on the WGCNA documentation, the SFT value was set to 8 to construct the co-expression network, and 11 modules were obtained (Fig. 3E, F). Genes in brown, yellow, and turquoise modules are related to disease occurrence (*P* < 0.05, Fig. 3G).

**Fig. 4:**
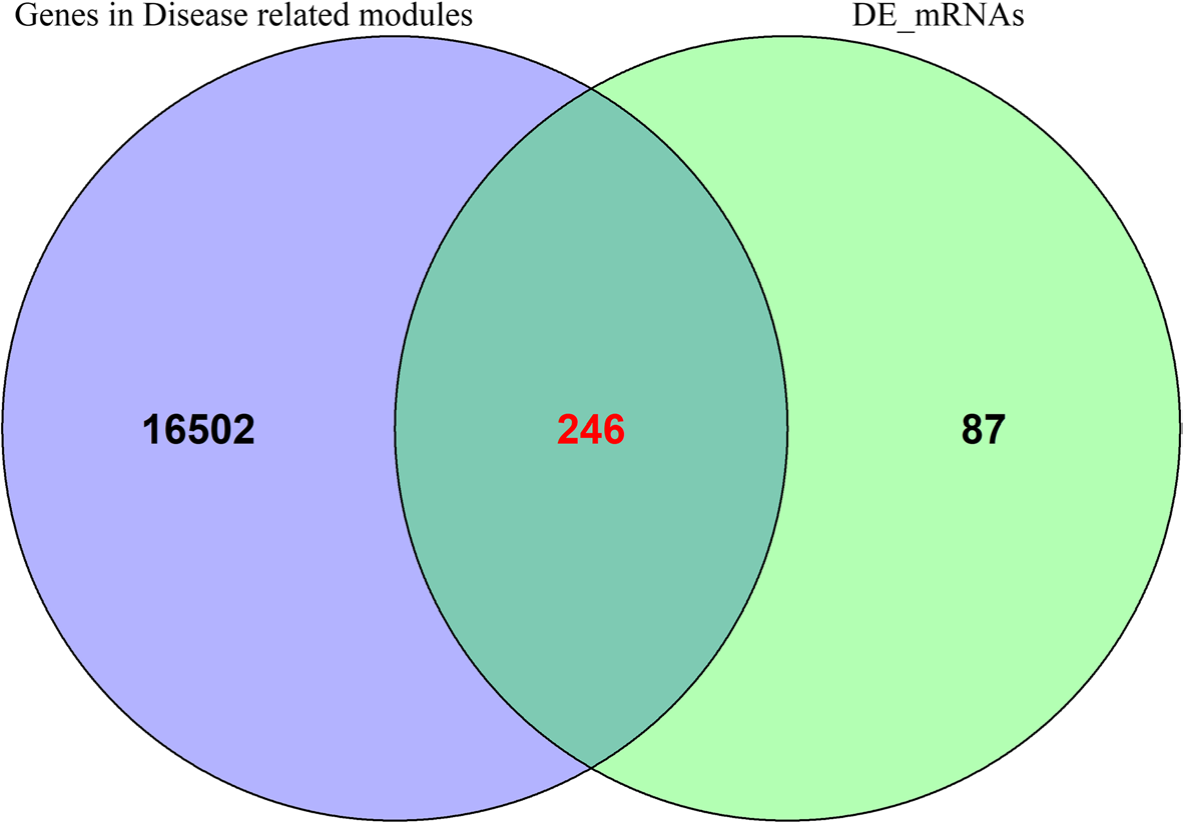
Venn diagram of joint analysis

A total of 246 DEGs were obtained from disease-related modules by exporting the genes from these three modules and combining them with differential expression analysis (Data collection Section) (Fig. 4).

### LncRNA

**Fig. 5:**
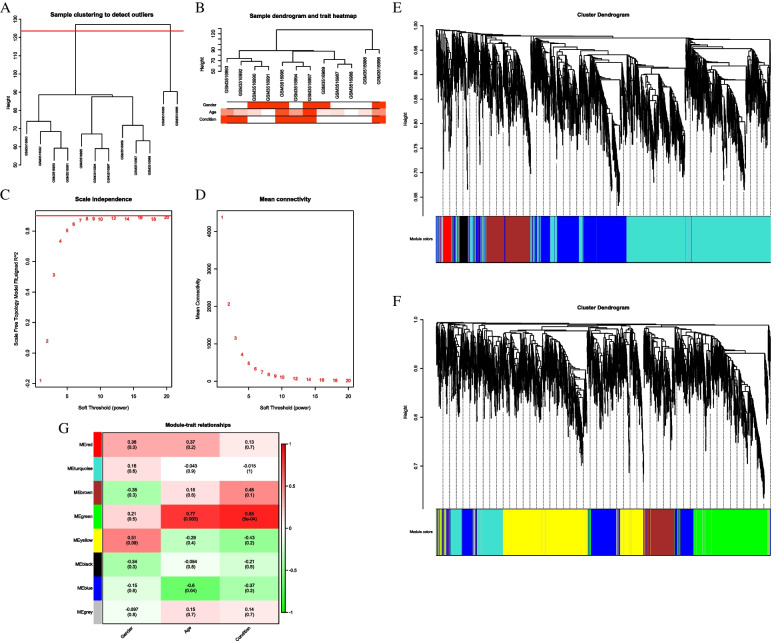
Sample cluster analysis (**A** and **B**); Calculation and selection of soft threshold (**C** and **D**); Cluster analysis of modules (**E** and **F**); Correlation analysis between modules and traits (**G**)

None of the 12 samples showed abnormal gene expression (Fig. 5A). Cluster analysis of gene expression profiles was performed by combining clinical information of samples (Fig. 5B). The experimental results converged when the SFT value was 8 (Figs. 5C, D). According to the WGCNA documentation, the SFT value was set to 7 to construct the co-expression network, and eight modules were obtained (Figs. 5E, F). Genes under the green module were significantly correlated with disease occurrence (*P* < 0.05, Fig. 5G).

**Fig. 6:**
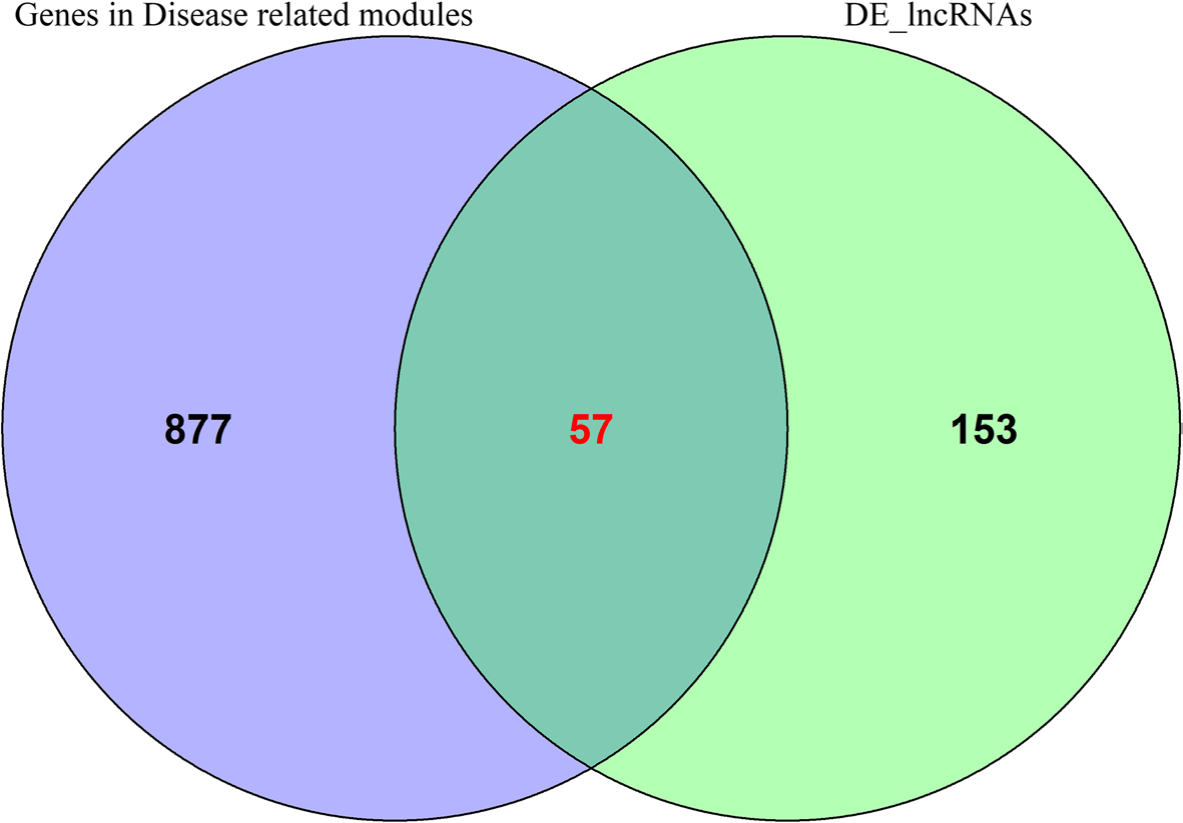
Venn diagram of joint analysis

The co-expressed genes in the green module were exported and combined with the differential expression analysis results of lncRNA (Data collection Section), which obtained 57 DEGs (Fig. 6).

### Competing endogenous RNA (ceRNA) network construction

#### LncRNA target gene prediction

StarBase was used to predict 57 lncRNAs target genes to obtain the predicted results of nine lncRNA (Table 1).

**Table 1:** Prediction results of some lncRNA target genes

miRNA name	gene name	ClipExp Num	miRseq	align	targetSeq
hsa-miR-520a-5p	PCBP1-AS1	1	ucuuucaugaaggGAGACCUc	|||||||	ggaaccucagugaCUCUGGAu
hsa-miR-525-5p	PCBP1-AS1	1	ucUUUCACGUAGGGAGACCUc	|| |:| |||||||	ggAACCUCAGUGACUCUGGAu
hsa-miR-3186-3p	AC100861.1	1	guUUCG-GUAGAGAGGCGCACu	|| | |: | | |||||||	ucAACCACGGC-CGCCGCGUGg
hsa-miR-873-5p	AC100861.1	3	uccucugaguGUUCAAGGACg	| ||||||||	uuucugugacCCAGUUCCUGg
hsa-miR-1245b-5	TEX41	1	aaAUUCACUAGAU-UUCCGGAu	||| ||: ||: |||||||	ugUAA-UGGCCUGCAAGGCCUu
hsa-miR-885-5p	TEX41	1	ucuccgucccaucACAUUACCu	||||||||	acaccaacauuuuUGUAAUGGc
hsa-miR-3142	TEX41	1	agAC-UUCCAAGUCUUUCCGGAa	|| || | | | |||||||	uuUGUAAUGGCCUGCAAGGCCUu
hsa-miR-216b-5p	AL355076.2	1	aguguaaaCGGACGUCUCUAAa	|:| ||||||||	uggaguaaGUCCACAGAGAUUc
hsa-miR-136-5p	AL355076.2	1	aggUAGUAGUUUU–-GUUUACCUCa	:|||| ||:::|||||||	ggaGUCAUAAAUGUUCUGAAUGGAGu

clipExpNum stands for experimental support. More detailed parameters can be found in the official website (http://starbase.sysu.edu.cn/index.php)

#### Prediction of miRNA target genes

StarBase was used to predict lncRNA target genes-miRNAs (Table 2).

**Table 2:** Prediction results of miRNAs target genes

miRNA name	gene name	geneID	chromosome	strand	clipExpNum
hsa-miR-103a-3p	DDX3Y	ENSG00000067048	chrY	+	10
hsa-miR-525-5p	SSU72	ENSG00000160075	chr1	-	8
hsa-miR-525-5p	SLC35E2	ENSG00000215790	chr1	-	1
hsa-miR-103a-3p	IRS1	ENSG00000169047	chr2	-	11
hsa-miR-103a-3p	COL4A4	ENSG00000081052	chr2	-	1
hsa-miR-107	ZNF629	ENSG00000102870	chr16	-	2
hsa-miR-107	C16orf58	ENSG00000140688	chr16	-	1
hsa-miR-873-5p	RCC2	ENSG00000179051	chr1	-	5
hsa-miR-873-5p	UBR4	ENSG00000127481	chr1	-	7
hsa-miR-873-5p	AKR7A2	ENSG00000053371	chr1	-	4

clipExpNum stands for experimental support. More detailed parameters can be found in the official website (http://starbase.sysu.edu.cn/index.php)

#### Establishment of the ceRNA regulatory network

**Fig. 7:**
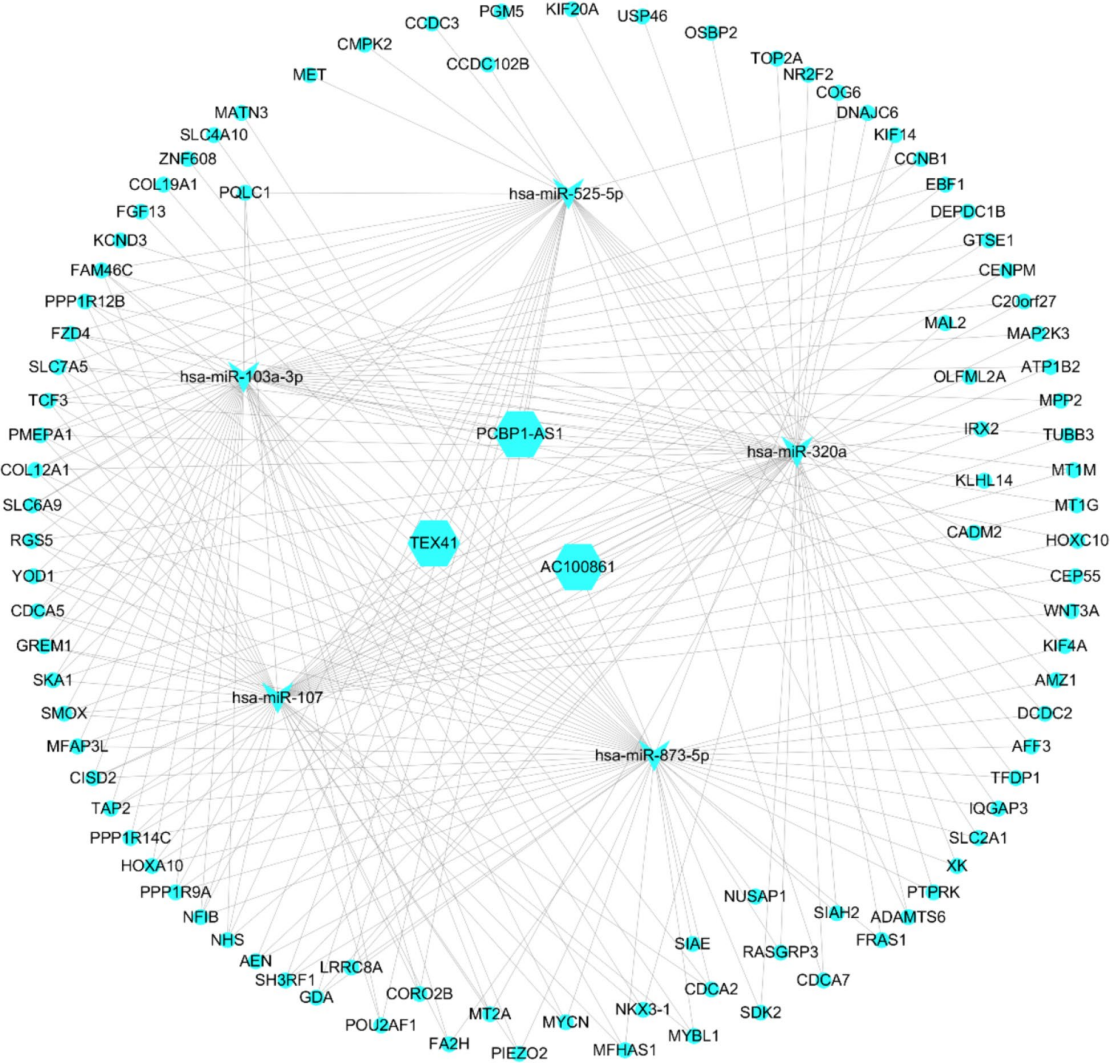
CeRNA network (hexagon: lncRNAs; inverted triangle: miRNAs; circle: mRNA)

Combined with the prediction results and difference analysis results, lncRNA-miRNA and miRNA-mRNA were identified, and Cytoscape software was used for ceRNA-network visualization (Fig. 7).

#### GO and KEGG pathway analyses

**Fig. 8:**
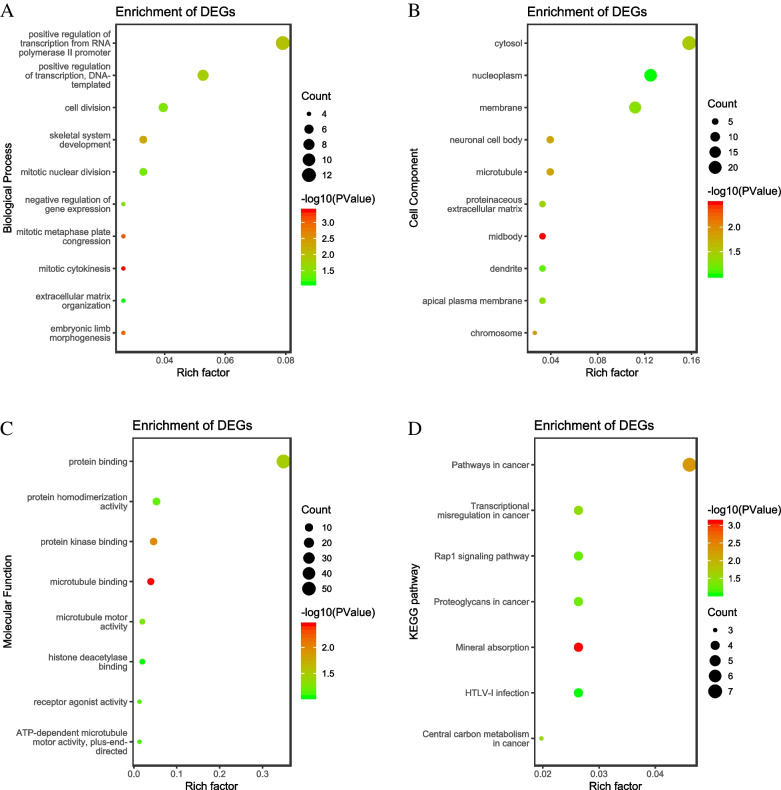
GO and KEGG enrichment analysis: (**A**) Cell component; (**B**) Molecular function; (**C**) biological process; and (**D**) KEGG pathway

GO and KEGG pathway analyses were performed on mRNA in the ceRNA network using DAVID, and the results were visualized using the R language (Fig. 8).

#### PPI analysis

**Fig. 9:**
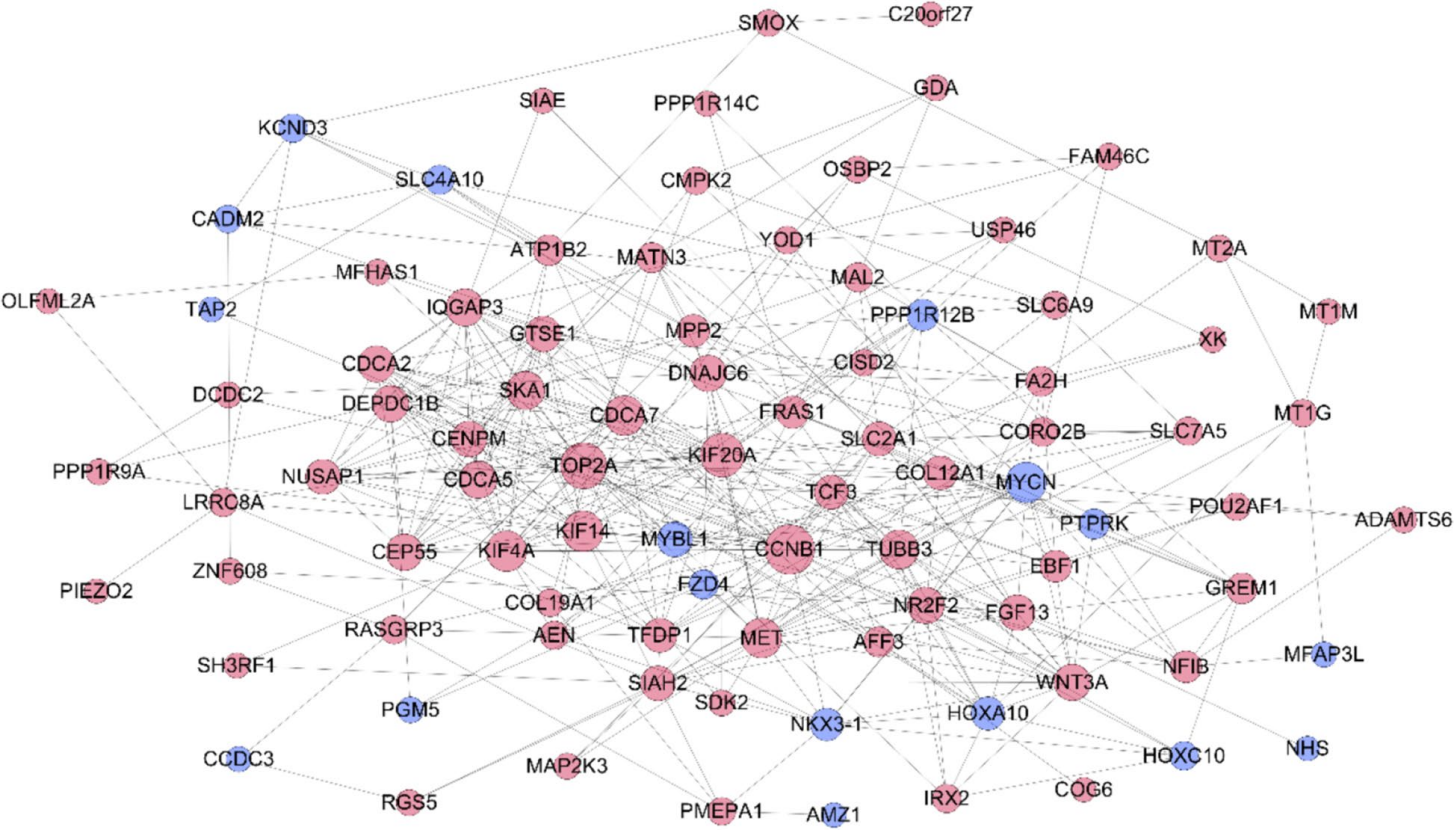
PPI (blue dots: low expression; red dots: high expression)

PPI analysis was performed for genes in the lncRNA-miRNA-mRNA regulatory network using STRING. The analysis results were visualized using Cytoscape (Fig. 9).

#### Hub gene validation

Using PPI network analysis of genes in the lncRNA-miRNA-mRNA regulatory network, the following hub genes were obtained (Table 3). The gene expression data in dataset GSE84044 were used to verify the expression of hub genes (genes with degree > 20 in the PPI network).

**Table 3:** Hub genes in the PPI network

Name	Degree	Stress	Average Shortest Path Length	Betweenness Centrality	Closeness Centrality
CCNB1	32	4718	1.753	0.135	0.571
TOP2A	28	2966	1.876	0.071	0.533
KIF20A	25	2134	2.000	0.045	0.500
KIF4A	21	1124	2.090	0.030	0.478
KIF14	21	1402	2.034	0.038	0.492
MYCN	21	2294	1.966	0.047	0.509
CDCA7	20	1428	2.034	0.027	0.492
MET	20	2692	2.000	0.064	0.500

**Fig. 10:**
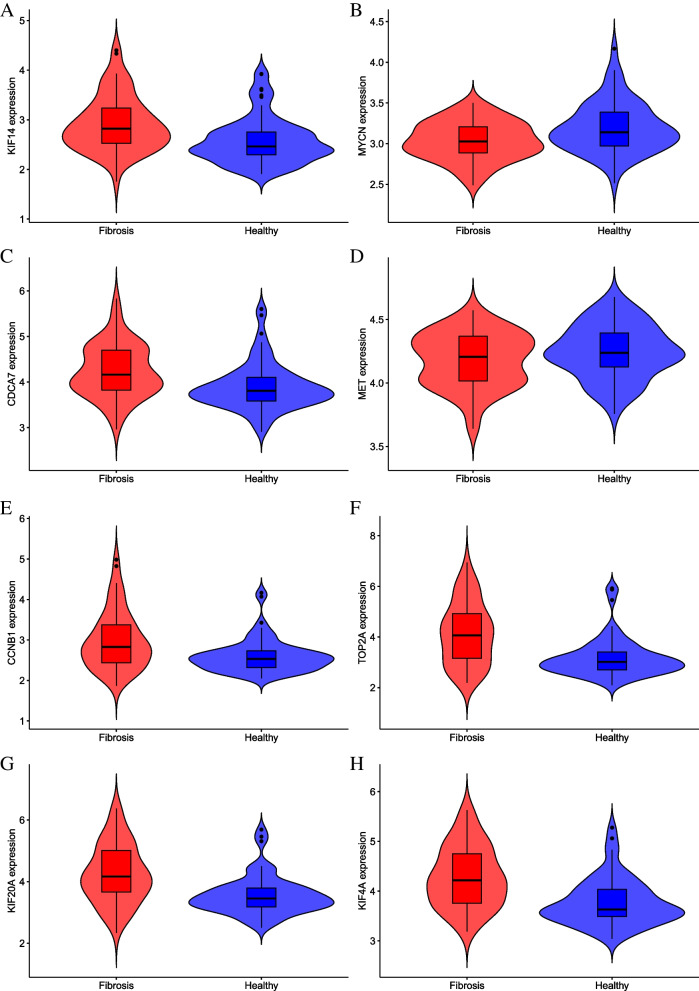
The expression of hub genes: **A**. CCNB1; **B**. TOP2A; **C**. KIF20A; **D**. KIF4A; **E**. KIF14; **F**. MYCN; **G**. CDCA7; **H**. MET

The expressions of hub genes in the eight cases were verified using GSE840044. The expression differences of genes (CCNB1, TOP2A, KIF20A, KIF4A, KIF14, MYCN, CDCA7) were found (except MET) (*p* < 0.05) (Fig. 10).

#### The downregulation of lncRNA AC100861

**Fig. 11:**
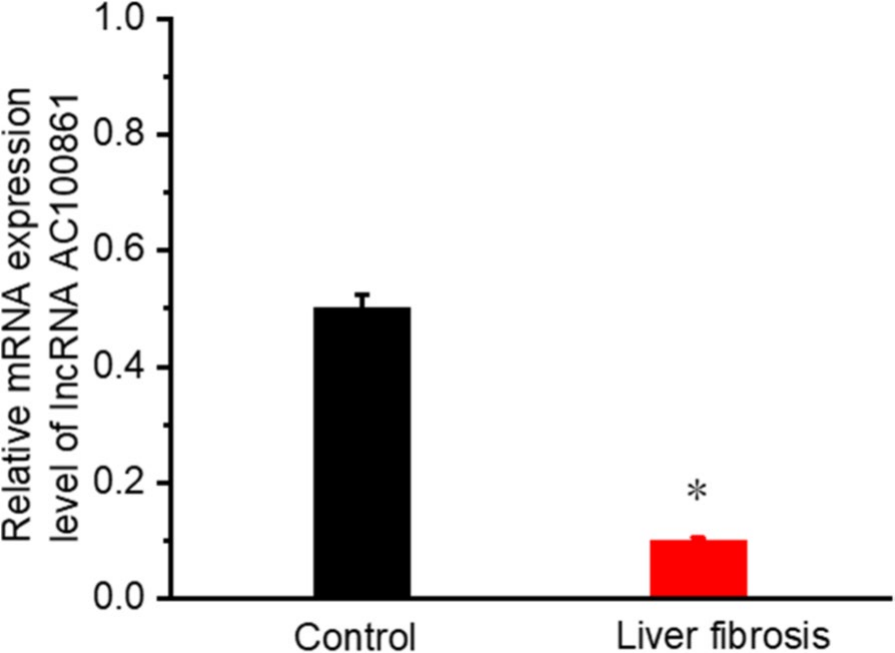
Downregulation of lncRNA AC100861 in liver fibrosis. All data are expressed as mean ± standard deviation (SD).The experiment was repeated three times. * *p* < 0.05, ** p < 0.01

To verify the critical lncRNAs involved in liver fibrosis, qRT-PCR was performed in fibrotic and healthy livers. LncRNA AC100861 mRNA expression was much lower in samples from liver fibrosis patients than healthy liver tissues (Fig. 11).

#### The downregulation of lncRNA AC100861 in activated HSCs

**Fig. 12:**
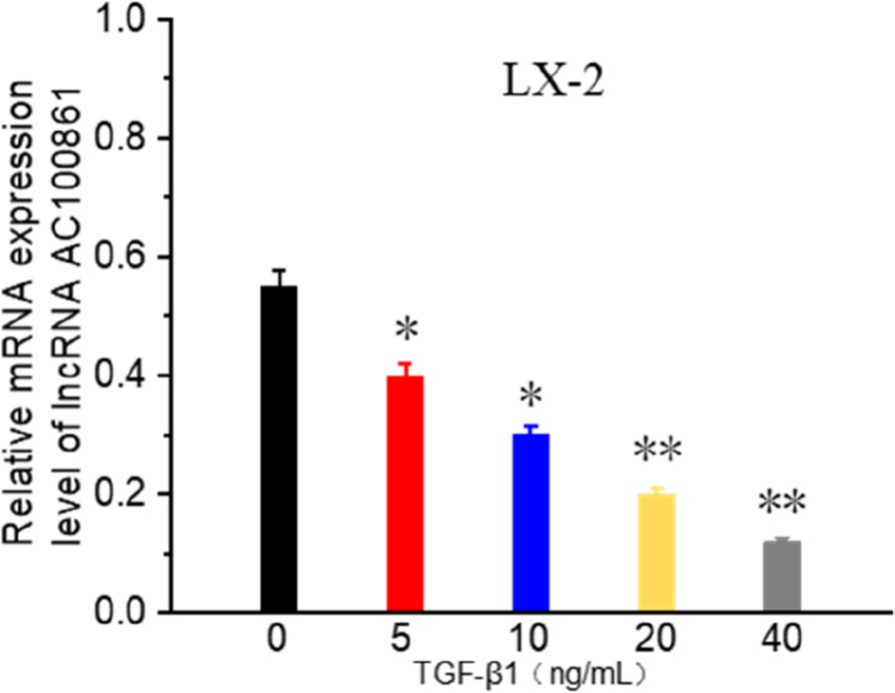
Downregulation of lncRNA AC100861 in activated HSCs. All data are expressed as mean ± standard deviation (SD).The experiment was repeated three times. * *p* < 0.05, ** *p* < 0.01

Because activated HSCs (LX-2) are thought to be the significant cellular participants in accelerating the deposition of ECM proteins, we determined whether lncRNA AC100861 participated in HSC activation. QRT-PCR analysis showed that compared with quiescent HSCs, lncRNA AC100861 expression was lower in activated HSCs treated with various concentrations of TGF-β1 (Fig. 12).

## Discussion

Liver fibrosis is considered a severe health problem worldwide. Liver fibrosis is an intense and reversible wound-healing process resulting from tissue necrosis [31, 32]. The resting HSCs transdifferentiate into myofibroblasts responsible for the deposition of ECM proteins (or collagen fibers), causing tissue scarring [7, 33]. Therefore, it is of great significance to study biomarkers for the diagnosis of liver fibrosis.

LncRNA, a small RNA molecule with 200 nucleotides, is closely involved in critical processes of cell development, proliferation, differentiation, and pluripotency [34]. With the improvement of deep transcriptome sequencing technology, the study of RNA molecules has significantly increased [35]. Although lncRNAs without protein-coding ability and bio-function, previous studies have provided ample evidence that these RNA molecules play a crucial function in controlling the expression of a gene by a series of mechanisms (such as targeted transcription) [36], affecting splicing function [37], targeting cis-acting promoter RNAs [38]. In recent decades, lncRNAs are involved in developing liver fibrosis [39–42].

LncRNAs act by regulating the binding of micro RNAs to targeted mRNA. The mechanism of co-expression and interaction between mRNA miRNAs and lncRNAs in disease regulation requires clarification. In this study, using the gene expression data from the GEO database and R language LIMMA package, we conducted bioinformatics mining on GSE12392 and GSE84044 datasets and analyzed the significantly expressed lncRNA and mRNA in tissues of patients with liver fibrosis. Then, 57 lncRNAs and 246 mRNAs were changed in liver fibrosis compared to the control group. Finally, we constructed the lncRNA-miRNA-mRNA network that included three lncRNAs, five miRNAs, and 93 mRNAs.

To further understand the effect of lncRNA activity on function, the possible biological mechanisms of lncRNA in liver fibrosis tissues were clarified through GO and KEGG. The analysis of GO functional enrichment were mainly manifested in cytosol, cytoplasm and other cellular components, protein binding, protein dimer activity, protein kinase binding and other molecular functions, as well as positive transcriptional regulation of DNA templatization and significant enhancement of extracellular matrix tissues. These results suggested that liver fibrosis-related enrichment of GO is mainly related to cell growth and tissue hyperplasia. KEGG pathway analysis revealed that this pathway is generally enriched in tumor transcriptional dysregulation, Rap1 signaling, tumor proteoglycan, mineral absorption, HTLVL infection and central carbon metabolism.

We found eight candidate hub genes using PPI analysis of genes in the lncRNA-miRNA-mRNA regulatory network using STRING. The expression of the eight hub genes was verified using GSE84044, and seven hub genes (CCNB1, TOP2A, KIF20A, KIF4A, KIF14, MYCN, CDCA7) (*p* < 0.05) showed significant differences between the groups (except MET). A study showed that CCNB1 and KIF20A are related to the activation of HSCs [43].

LncRNAs with more edges are hubs that participate in more ceRNA interactions, suggesting that lncRNAs participate in network organization. According to node degree and relationship, we selected the critical lncRNAs (TEX41, PCBP1-AS1, and AC100861). These lncRNAs can be used as biomarkers for the diagnosis of liver fibrosis. To speculate about the potential functions of lncRNAs, the function of annotated miRNAs or mRNAs can be studied based on the ceRNA theory. LncRNA-miRNA-mRNA can offer a holistic view of the ceRNA interactions, which permit the study of the regulatory properties of lncRNAs; however, the subnetwork of the key lncRNA-miRNA-mRNA detailed how key lncRNAs work in conjunction with competing mRNAs [44]. A growing body of evidence suggests that lncRNAs and miRNAs ‘chat’ with one another in ceRNA processes [45]. In the present study case, lncRNAs may act as sponges to isolate miRNAs from their targeted mRNAs, causing changes in the expression of their target genes [46]. The CeRNA network showed that DE lncRNAs in liver fibrosis bound to miR-103a-3p, miR-525-5p, miR-103a-3p, miR-107, and miR-873-5p, can effectively regulate a large of target genes. Of the three lncRNAs, some may competitively bind to these miRNAs through the ceRNA affect and regulate the expression of hub genes (ACCNB1, TOP2A, kif20a, dkif4a, kif14, MYCN, and CDH2).

QRT-PCR results indicated no significant differences in expression of lncRNA TEX41 or PCBP1-AS1 between liver fibrosis tissue control liver tissue; however, the mRNA level of lncRNA AC100861 (lncRNA TNFRSF10A-DT) was significantly decreased. At present, there are no reports on the correlation between the lncRNA TNFRSF10A-DT and liver fibrosis. The mRNA expression of lncRNA TNFRSF10A-DT in activated HSCs was significantly lower than in resting HSCs. Our study demonstrated that the lncRNA TNFRSF10A-DT expression is correlated with liver fibrosis and may be helpful for diagnosis and outcome prediction.

We used StarBase to predict the interactions between lncRNA and miRNA and found that the predicted target miRNA of lncRNA TNFRSF10A-DT was miR-873–5. Next, we used StarBase to obtain miRNA-targeted mRNAs and found that the predicted target genes of miR-873–5 were RCC2, UBR4 and AKR7A2. Based on the ceRNA network, lncRNA TNFRSF10A-DT/has-mir-873–5/RCC2, UBR4, and the AKR7A2 axis may construct a ceRNA network to participate in the progression of liver fibrosis.

In summary, we constructed a liver fibrosis-related lncRNA-miRNA-mRNA network to study the biological functions of lncRNA in liver fibrosis development according to ceRNA theory. LncRNA TNFRSF10A-DT was the critical lncRNA in the ceRNA network. QRT-PCR indicated that the mRNA level of lncRNA TNFRSF10A-DT in liver fibrosis patients and the expression level of activated HSCs were much lower than in healthy people and resting HSCs. These findings suggest that lncRNA TNFRSF10A-DT may serve as a biomarker and therapeutic target for liver fibrosis. We speculate that lncRNA TNFRSF10A-DT may compete with miR-873–5 to regulate the target mRNA genes (RCC2, UBR2, and AKR7A2) in the ceRNA network. The ceRNA network is helpful to improve understanding of the pathogenesis of liver fibrosis. However, the specific ceRNA mechanism of lncRNA TNFRSF10A-DT requires further research.

### Supplementary Information

**Additional file 1:** **Table S1.** The expression data of mRNA and lncRNA in GSE123932.

## Data Availability

The datasets generated and/or analyzed during the current study are available in the Gene Expression Omnibus (GEO) datasets (accession no. GSE12392 and GSE84044; https://www.ncbi.nlm.nih.gov/geo/query/acc.cgi?acc=GSE12392; https://www.ncbi.nlm.nih.gov/geo/query/acc.cgi?acc = GSE84044).

## Abbreviations

DEGs: Diferentially expressed genes
GEO: Gene Expression Omnibus
GO: Gene Ontology
KEGG: Kyoto Encyclopedia of Genes and Genomes
PPI: Protein–protein interaction
CC: Cellular component
MF: Molecular function
BP: Biological process
STRING: Search Tool for Retrieval of interacting Genes
WGCNA: Weighted Gene Co-expression Network Analysis
qRT-PCR: Quantitative Real-Time PCR
