# Supplementary material for: Identification of functional genes in liver fibrosis based on bioinformatics analysis of a lncRNA-mediated ceRNA network
Source: BMC Med Genomics. 2024 Feb 20;17:56. doi: 10.1186/s12920-024-01813-x (PMC10877760; doi:10.1186/s12920-024-01813-x)
Supplement: Supplementary file 1 — Additional file 1: Table S1. The expression data of mRNA and lncRNA in GSE123932. [file 12920_2024_1813_MOESM1_ESM.docx]

**Supporting Information**

Table S1 The expression data of mRNA and lncRNA in GSE123932

| ID | logFC | AveExpr | t | P.Value | adj.P.Value | B | GeneSymbol | type |
| --- | --- | --- | --- | --- | --- | --- | --- | --- |
| A_19_P00317277 | -1.226879 | 4.374774046 | -3.14572 | 0.007811571 | 0.265869094 | -2.318768 | LOC100506328 | mRNA |
| A_19_P00318609 | 1.7676656 | 2.610114842 | 4.810389 | 0.000349231 | 0.174988778 | 0.3572038 | P39189 | mRNA |
| A_19_P00319213 | -1.180028 | 3.319590817 | -2.43855 | 0.030000282 | 0.379600972 | -3.482224 | XLOC_010889 | mRNA |
| A_21_P0000076 | 1.3218362 | 2.393130986 | 4.323261 | 0.000843979 | 0.202947597 | -0.392148 | CDK1 | mRNA |
| A_21_P0000190 | 1.1749224 | 6.165877311 | 2.519469 | 0.025772515 | 0.36022958 | -3.352115 | AFF3 | mRNA |
| A_21_P0001030 | -1.312695 | 4.726270053 | -4.34832 | 0.00080597 | 0.199930261 | -0.352726 | XLOC_000468 | mRNA |
| A_21_P0001053 | 1.0058354 | 3.029674309 | 3.674353 | 0.002844194 | 0.226472045 | -1.440132 | XLOC_000545 | mRNA |
| A_21_P0001519 | 1.1654713 | 2.503467918 | 4.320373 | 0.000848476 | 0.202947597 | -0.396697 | XLOC_000877 | mRNA |
| A_21_P0001770 | 1.5384261 | 3.127971426 | 3.129841 | 0.008053025 | 0.266991578 | -2.345251 | XLOC_001362 | mRNA |
| A_21_P0003888 | -1.050275 | 4.613409339 | -2.30694 | 0.038331918 | 0.407809322 | -3.691032 | XLOC_003870 | mRNA |
| A_21_P0003975 | -2.170519 | 3.78681504 | -6.08545 | 0.0000403 | 0.113876128 | 2.1279054 | XLOC_004309 | mRNA |
| A_21_P0004640 | -1.16457 | 2.771605013 | -3.23235 | 0.00661605 | 0.25593825 | -2.174233 | XLOC_005239 | mRNA |
| A_21_P0004641 | 1.2666394 | 1.949331197 | 5.07019 | 0.000220839 | 0.158611722 | 0.7411579 | MUC22 | mRNA |
| A_21_P0004702 | 1.2208377 | 2.401923683 | 3.788832 | 0.00228945 | 0.226472045 | -1.25198 | XLOC_005529 | mRNA |
| A_21_P0004765 | -3.583764 | 3.436148633 | -3.06749 | 0.009075545 | 0.273473567 | -2.44921 | XLOC_005780 | mRNA |
| A_21_P0005118 | -1.262711 | 2.761374006 | -3.07047 | 0.009023857 | 0.273473567 | -2.444244 | XLOC_005361 | mRNA |
| A_21_P0005235 | -1.115376 | 3.087245721 | -2.46937 | 0.028316984 | 0.374406941 | -3.432819 | XLOC_006105 | mRNA |
| A_21_P0005333 | -1.306595 | 4.039450773 | -2.18972 | 0.04756442 | 0.431329696 | -3.873523 | XLOC_006596 | mRNA |
| A_21_P0005390 | -1.397522 | 2.259809448 | -3.11589 | 0.008271329 | 0.268397133 | -2.368517 | LOC285902 | mRNA |
| A_21_P0005955 | -1.887656 | 2.723110336 | -3.60845 | 0.00322388 | 0.226595231 | -1.548931 | XLOC_006938 | mRNA |
| A_21_P0006454 | 1.0430057 | 7.528498052 | 4.466343 | 0.000649356 | 0.1889821 | -0.168277 | FLJ46446 | mRNA |
| A_21_P0006626 | -1.083635 | 3.921206228 | -2.29424 | 0.039243653 | 0.410027764 | -3.710978 | XLOC_008916 | mRNA |
| A_21_P0007131 | 1.2297783 | 2.796677576 | 4.997974 | 0.000250617 | 0.162553732 | 0.6355827 | XLOC_009323 | mRNA |
| A_21_P0008119 | 1.1247241 | 2.373328491 | 2.822179 | 0.014512862 | 0.306211164 | -2.856738 | XLOC_010500 | mRNA |
| A_21_P0008122 | 1.0459923 | 2.594392201 | 2.880098 | 0.012992878 | 0.298863997 | -2.760843 | XLOC_010503 | mRNA |
| A_21_P0008258 | 1.2254853 | 4.130529986 | 4.35348 | 0.000798365 | 0.199930261 | -0.344619 | XLOC_010640 | mRNA |
| A_21_P0008272 | 1.4392123 | 3.796923504 | 5.327992 | 0.000141408 | 0.150068671 | 1.1106579 | XLOC_010917 | mRNA |
| A_21_P0009267 | 1.3284376 | 2.522808362 | 2.902685 | 0.012443673 | 0.294795504 | -2.723379 | XLOC_012291 | mRNA |
| A_21_P0009422 | -1.162522 | 7.116066745 | -3.54621 | 0.003629663 | 0.231517872 | -1.651942 | XLOC_012669 | mRNA |
| A_21_P0009673 | -1.18235 | 8.131092006 | -3.92607 | 0.001767421 | 0.226017267 | -1.028031 | XLOC_013318 | mRNA |
| A_21_P0009957 | -1.103165 | 9.225595397 | -2.80379 | 0.015031272 | 0.307861674 | -2.887135 | XLOC_013531 | mRNA |
| A_21_P0010144 | 1.0048222 | 3.296835309 | 2.993084 | 0.010466434 | 0.282851253 | -2.573142 | XLOC_013874 | mRNA |
| A_21_P0010977 | 1.2060384 | 7.34581714 | 3.685635 | 0.002783912 | 0.226472045 | -1.421541 | GLYATL1 | mRNA |
| A_21_P0011347 | 1.0962661 | 11.12954032 | 2.744227 | 0.016837922 | 0.319097499 | -2.985346 | XLOC_l2_004840 | mRNA |
| A_21_P0012599 | -2.463986 | 3.663739241 | -4.08773 | 0.001305776 | 0.21218118 | -0.766855 | XLOC_l2_010724 | mRNA |
| A_21_P0012947 | -1.114372 | 4.463523981 | -3.5032 | 0.003940085 | 0.234467777 | -1.723282 | XLOC_l2_011692 | mRNA |
| A_21_P0013462 | 1.3330814 | 2.682806878 | 4.401506 | 0.000731049 | 0.198510187 | -0.269355 | XLOC_l2_014077 | mRNA |
| A_21_P0013507 | -1.087194 | 7.423845146 | -3.78143 | 0.002321751 | 0.226472045 | -1.264119 | XLOC_l2_014423 | mRNA |
| A_21_P0013740 | 1.1540044 | 3.99889108 | 3.084493 | 0.008784534 | 0.271258138 | -2.420871 | XLOC_l2_015315 | mRNA |
| A_21_P0013821 | 1.0672233 | 4.199970257 | 2.834024 | 0.014188385 | 0.304651653 | -2.837148 | XLOC_l2_015764 | mRNA |
| A_21_P0014082 | -1.083934 | 3.606653661 | -2.59688 | 0.022270155 | 0.343223376 | -3.226588 | LOC100507013 | mRNA |
| A_21_P0014582 | -1.380949 | 2.946203565 | -2.8394 | 0.014043566 | 0.303932121 | -2.828258 | LOC100507330 | mRNA |
| A_23_P101093 | -1.226881 | 3.650214861 | -2.75529 | 0.016486939 | 0.316358114 | -2.96713 | COPZ2 | mRNA |
| A_23_P102058 | 1.3414798 | 2.21640091 | 3.069625 | 0.009038533 | 0.273473567 | -2.445657 | MATN3 | mRNA |
| A_23_P102731 | 1.3724249 | 9.582146263 | 3.339114 | 0.005392139 | 0.246716909 | -1.996213 | SMOX | mRNA |
| A_23_P104651 | 1.117097 | 2.80095876 | 2.570591 | 0.0234045 | 0.349549204 | -3.269327 | CDCA5 | mRNA |
| A_23_P106844 | 1.2103081 | 10.80328149 | 4.112825 | 0.0012461 | 0.208880693 | -0.726582 | MT2A | mRNA |
| A_23_P107735 | 1.0770841 | 10.05835471 | 3.693276 | 0.002743823 | 0.226472045 | -1.408955 | CD79A | mRNA |
| A_23_P113748 | -1.552077 | 4.228045127 | -4.2069 | 0.001046283 | 0.204841282 | -0.576341 | ZNF385D | mRNA |
| A_23_P114713 | 1.3348577 | 2.125001884 | 5.700467 | 0.0000755 | 0.132760966 | 1.6237162 | CYP4B1 | mRNA |
| A_23_P114947 | -1.073212 | 12.26224679 | -2.8554 | 0.013620812 | 0.301767151 | -2.801767 | RGS2 | mRNA |
| A_23_P115872 | 1.7883674 | 3.395870147 | 4.60032 | 0.00050913 | 0.183230844 | 0.0385725 | CEP55 | mRNA |
| A_23_P117694 | 1.3351786 | 3.879113539 | 2.66241 | 0.019670721 | 0.330234658 | -3.119642 | CORO2B | mRNA |
| A_23_P117852 | 1.292672 | 6.150871167 | 3.353575 | 0.005244895 | 0.243331186 | -1.97212 | KIAA0101 | mRNA |
| A_23_P118834 | 1.017638 | 4.424643948 | 4.920058 | 0.000287489 | 0.165068971 | 0.5206738 | TOP2A | mRNA |
| A_23_P121480 | 1.0603025 | 7.704575605 | 3.323004 | 0.005561086 | 0.248634337 | -2.023061 | CD200 | mRNA |
| A_23_P122197 | 1.6235112 | 4.729863727 | 3.869157 | 0.00196729 | 0.226017267 | -1.120674 | CCNB1 | mRNA |
| A_23_P123228 | 1.0316278 | 3.435765423 | 2.766632 | 0.016134571 | 0.313104747 | -2.948442 | SLC26A3 | mRNA |
| A_23_P123488 | 1.0563762 | 4.3153703 | 6.267788 | 0.0000301 | 0.11060151 | 2.3574697 | PRDM14 | mRNA |
| A_23_P12405 | -1.515707 | 5.035281148 | -3.17073 | 0.007445808 | 0.260974545 | -2.277046 | ESPN | mRNA |
| A_23_P124417 | 1.2592898 | 4.558849933 | 2.752738 | 0.016567271 | 0.317084071 | -2.971334 | BUB1 | mRNA |
| A_23_P127662 | 1.1112506 | 3.014943148 | 2.172367 | 0.049097381 | 0.435500582 | -3.900222 | OR8D1 | mRNA |
| A_23_P130376 | 1.0555817 | 3.094934854 | 5.478391 | 0.000109487 | 0.140344925 | 1.320803 | PIEZO2 | mRNA |
| A_23_P131060 | -1.004872 | 9.033903118 | -3.26092 | 0.006263491 | 0.252302678 | -2.126576 | CYP4F8 | mRNA |
| A_23_P13195 | 1.2870539 | 4.146305049 | 5.134021 | 0.000197594 | 0.15253467 | 0.8337264 | OR8G1 | mRNA |
| A_23_P132956 | 1.1441126 | 1.746594781 | 5.718847 | 0.0000732 | 0.132760966 | 1.6483906 | UCHL1 | mRNA |
| A_23_P133842 | -1.26679 | 5.658684523 | -3.13446 | 0.007981981 | 0.266991578 | -2.337542 | HIST1H1T | mRNA |
| A_23_P137665 | -1.571381 | 8.994730751 | -2.94832 | 0.011403249 | 0.289435494 | -2.647596 | CHI3L1 | mRNA |
| A_23_P139654 | -1.113025 | 6.101264366 | -2.78351 | 0.015623794 | 0.310524041 | -2.920605 | KLRC1 | mRNA |
| A_23_P140342 | 1.2317741 | 4.525623244 | 3.898665 | 0.001860933 | 0.226017267 | -1.0726 | FSCB | mRNA |
| A_23_P14351 | 1.4008054 | 3.918167052 | 2.629842 | 0.020923428 | 0.335868481 | -3.172869 | AKAP6 | mRNA |
| A_23_P143902 | -1.338741 | 6.170429374 | -3.63968 | 0.003037903 | 0.226595231 | -1.497329 | P2RY12 | mRNA |
| A_23_P154338 | -1.02826 | 2.304840845 | -3.14869 | 0.007767151 | 0.265314178 | -2.313807 | EFHD1 | mRNA |
| A_23_P155786 | -1.923701 | 2.717993489 | -2.9413 | 0.011557501 | 0.289969093 | -2.659262 | SULT1E1 | mRNA |
| A_23_P156025 | 1.198738 | 2.182451187 | 4.286777 | 0.000902656 | 0.203593727 | -0.449701 | IRX2 | mRNA |
| A_23_P159406 | 1.0577243 | 2.134304125 | 3.505577 | 0.003922219 | 0.234467777 | -1.71933 | SPRR1B | mRNA |
| A_23_P169978 | 1.3210775 | 3.601582538 | 2.465382 | 0.028529541 | 0.374982761 | -3.439221 | ZNF608 | mRNA |
| A_23_P17134 | 1.0599365 | 9.302637002 | 4.26205 | 0.000944805 | 0.203612481 | -0.488812 | MAL | mRNA |
| A_23_P17481 | 1.5872912 | 5.189924228 | 2.828613 | 0.014335718 | 0.305172018 | -2.846099 | SIGLEC1 | mRNA |
| A_23_P17663 | 1.0675513 | 9.804693545 | 2.323961 | 0.037141906 | 0.403830987 | -3.664248 | MX1 | mRNA |
| A_23_P201790 | -1.201311 | 8.108842429 | -5.08732 | 0.000214335 | 0.158611722 | 0.7660625 | PPP1R12B | mRNA |
| A_23_P202881 | -1.074973 | 4.175061683 | -2.34608 | 0.035648055 | 0.398890177 | -3.629344 | FEZ1 | mRNA |
| A_23_P205428 | 1.118039 | 3.568619569 | 3.097286 | 0.008571707 | 0.271135093 | -2.399542 | FOXG1 | mRNA |
| A_23_P206077 | 1.0139044 | 5.117427345 | 2.726746 | 0.017407485 | 0.321507336 | -3.014103 | AEN | mRNA |
| A_23_P213319 | 1.564628 | 2.40233203 | 3.545778 | 0.00363267 | 0.231517872 | -1.652662 | ADAMTS6 | mRNA |
| A_23_P215744 | 1.0675562 | 4.340799092 | 3.505494 | 0.003922845 | 0.234467777 | -1.719469 | CTTNBP2 | mRNA |
| A_23_P216282 | 1.8737949 | 4.430346295 | 2.240966 | 0.043295852 | 0.421558149 | -3.794185 | ARHGEF10 | mRNA |
| A_23_P217319 | 1.0188671 | 4.869543843 | 2.934806 | 0.011702006 | 0.290875671 | -2.670048 | FGF13 | mRNA |
| A_23_P218442 | -1.585101 | 3.34491945 | -2.54618 | 0.024507663 | 0.35473779 | -3.30891 | CEACAM6 | mRNA |
| A_23_P22565 | 1.1608014 | 2.924300132 | 2.827438 | 0.014367912 | 0.305557106 | -2.848042 | FATE1 | mRNA |
| A_23_P23829 | -1.533049 | 2.24875576 | -3.88132 | 0.001922712 | 0.226017267 | -1.100846 | CD34 | mRNA |
| A_23_P24616 | 1.0606425 | 7.196681849 | 3.93379 | 0.001741964 | 0.226017267 | -1.015494 | SIAE | mRNA |
| A_23_P250245 | 1.0190695 | 7.888058943 | 2.568647 | 0.023490576 | 0.349740826 | -3.272483 | CD72 | mRNA |
| A_23_P250274 | 1.1804936 | 7.560521072 | 4.268911 | 0.00093291 | 0.203593727 | -0.477952 | LRRC8A | mRNA |
| A_23_P251421 | 1.2020937 | 3.490512087 | 3.236647 | 0.006561817 | 0.255276642 | -2.16707 | CDCA7 | mRNA |
| A_23_P254688 | -1.050544 | 3.555782516 | -2.97991 | 0.010733882 | 0.284987776 | -2.595061 | TMEM108 | mRNA |
| A_23_P254944 | 1.493571 | 6.159192686 | 5.332641 | 0.000140287 | 0.150068671 | 1.1172139 | GSTT1 | mRNA |
| A_23_P256956 | 1.0307303 | 5.031652505 | 2.456955 | 0.028983909 | 0.376638305 | -3.452745 | KIF20A | mRNA |
| A_23_P258912 | -2.866899 | 7.692029183 | -2.92769 | 0.011862376 | 0.291727186 | -2.681863 | MYOM2 | mRNA |
| A_23_P259707 | 1.0572741 | 3.255441204 | 3.10245 | 0.008487256 | 0.270600028 | -2.390931 | LPPR1 | mRNA |
| A_23_P2674 | -1.062632 | 4.202605142 | -2.20384 | 0.04635017 | 0.428690263 | -3.851732 | KRT4 | mRNA |
| A_23_P27528 | -1.050369 | 5.128235856 | -5.05001 | 0.000228767 | 0.158611722 | 0.7117527 | CYP2A7 | mRNA |
| A_23_P302550 | -1.204619 | 10.13353093 | -4.12162 | 0.001225856 | 0.208880693 | -0.712487 | RGS18 | mRNA |
| A_23_P302672 | 1.0909026 | 2.088880268 | 3.310096 | 0.005700291 | 0.248680669 | -2.044578 | DDIT4L | mRNA |
| A_23_P312920 | 1.1227986 | 8.060138303 | 3.4106 | 0.00470268 | 0.241999872 | -1.877171 | POU2AF1 | mRNA |
| A_23_P313550 | -1.042683 | 2.913224861 | -3.60257 | 0.003260143 | 0.226595231 | -1.558647 | SLC25A41 | mRNA |
| A_23_P31376 | -1.042645 | 8.171569153 | -4.17158 | 0.001117126 | 0.20577592 | -0.632617 | LRRN3 | mRNA |
| A_23_P315964 | 1.2548591 | 3.427725627 | 2.992598 | 0.010476199 | 0.282851253 | -2.573952 | UMODL1 | mRNA |
| A_23_P316501 | -1.024062 | 2.063982893 | -3.53641 | 0.003698119 | 0.231756767 | -1.668183 | NKAIN2 | mRNA |
| A_23_P323272 | 1.393693 | 5.355861658 | 3.249952 | 0.00639659 | 0.253549157 | -2.144876 | OSR1 | mRNA |
| A_23_P326760 | -1.076868 | 4.106869862 | -2.54483 | 0.024570278 | 0.355062945 | -3.311102 | MYRIP | mRNA |
| A_23_P327551 | -1.162624 | 2.507423145 | -4.622 | 0.000489574 | 0.182328187 | 0.0717888 | CPNE4 | mRNA |
| A_23_P350591 | -1.183891 | 2.839749571 | -2.66191 | 0.019689347 | 0.330234658 | -3.120458 | BEND2 | mRNA |
| A_23_P35066 | -1.231182 | 3.456456817 | -2.77972 | 0.015737095 | 0.310697631 | -2.926859 | SNX7 | mRNA |
| A_23_P357717 | 1.575432 | 8.874540497 | 3.867877 | 0.001972045 | 0.226017267 | -1.122763 | TCL1A | mRNA |
| A_23_P359245 | 1.3049406 | 3.207970285 | 3.635821 | 0.003060281 | 0.226595231 | -1.503702 | MET | mRNA |
| A_23_P361419 | 1.0153068 | 3.875248027 | 4.795734 | 0.000358471 | 0.174988778 | 0.3352075 | DEPDC1B | mRNA |
| A_23_P362759 | -1.252602 | 5.418079482 | -2.74453 | 0.016828107 | 0.319072281 | -2.984842 | PRDM5 | mRNA |
| A_23_P363174 | 1.3177718 | 8.725585702 | 3.38073 | 0.004979243 | 0.24210451 | -1.926891 | HIST1H2AL | mRNA |
| A_23_P363301 | 1.0374843 | 3.521061605 | 3.448107 | 0.004377257 | 0.23738443 | -1.814788 | PDCL2 | mRNA |
| A_23_P364414 | 1.2456055 | 4.856763677 | 3.880093 | 0.001927165 | 0.226017267 | -1.102847 | PCDHGA9 | mRNA |
| A_23_P36531 | 1.1433402 | 3.075104248 | 2.744374 | 0.016833203 | 0.319088474 | -2.985104 | TSPAN8 | mRNA |
| A_23_P368067 | -1.116316 | 6.890349537 | -2.44642 | 0.029561854 | 0.378131083 | -3.469635 | TAP2 | mRNA |
| A_23_P370830 | 1.2739786 | 3.771236193 | 2.667793 | 0.019470828 | 0.329735837 | -3.110831 | KLHL14 | mRNA |
| A_23_P375922 | 1.4233098 | 6.764594095 | 3.407965 | 0.004726447 | 0.241999872 | -1.881557 | COL19A1 | mRNA |
| A_23_P383258 | 1.0823211 | 4.976424763 | 4.43949 | 0.00068198 | 0.191826429 | -0.210065 | GDA | mRNA |
| A_23_P385690 | 1.0058247 | 3.286945062 | 3.29108 | 0.005911788 | 0.249862515 | -2.076283 | WNT3A | mRNA |
| A_23_P38584 | 1.1339089 | 2.842133251 | 3.659341 | 0.002926472 | 0.226472045 | -1.464885 | KRT27 | mRNA |
| A_23_P385861 | 1.4280165 | 2.786900315 | 4.980356 | 0.000258499 | 0.162928093 | 0.6096895 | CDCA2 | mRNA |
| A_23_P386254 | 1.0271491 | 2.489523991 | 2.584894 | 0.022780642 | 0.345533874 | -3.24609 | NKX3-2 | mRNA |
| A_23_P394972 | -1.741518 | 3.234179373 | -6.81466 | 0.0000129 | 0.081134697 | 3.0111076 | TSPEAR | mRNA |
| A_23_P401675 | -1.1615 | 5.205123593 | -2.6708 | 0.019359929 | 0.329735837 | -3.105902 | MARVELD2 | mRNA |
| A_23_P403443 | 1.2592203 | 4.20555941 | 4.085147 | 0.001312073 | 0.21218118 | -0.770998 | B4GALNT3 | mRNA |
| A_23_P408249 | 1.0562819 | 1.966757261 | 4.149607 | 0.00116367 | 0.206406666 | -0.667706 | PCK1 | mRNA |
| A_23_P422724 | 1.0900837 | 6.133102955 | 2.72093 | 0.017601135 | 0.322750794 | -3.023664 | PPIC | mRNA |
| A_23_P432947 | 1.7645621 | 2.919433194 | 3.087327 | 0.008736941 | 0.271258138 | -2.416147 | GREM1 | mRNA |
| A_23_P43415 | -1.688274 | 3.846741845 | -4.08698 | 0.001307589 | 0.21218118 | -0.76805 | HSD17B3 | mRNA |
| A_23_P45011 | 1.0799872 | 3.097725602 | 3.650038 | 0.00297867 | 0.226538548 | -1.480233 | PPP1R14C | mRNA |
| A_23_P45304 | 1.5011414 | 7.120952328 | 3.192197 | 0.00714551 | 0.259037125 | -2.241226 | XK | mRNA |
| A_23_P45871 | 1.4619569 | 8.638380195 | 2.234321 | 0.043828214 | 0.422709144 | -3.804513 | IFI44L | mRNA |
| A_23_P45976 | 1.5669739 | 8.8487339 | 2.782285 | 0.01566037 | 0.310524041 | -2.922629 | RAP1GAP | mRNA |
| A_23_P47728 | -1.067034 | 2.362028218 | -3.19894 | 0.00705377 | 0.258164555 | -2.229982 | MAP6 | mRNA |
| A_23_P49155 | 1.2032932 | 3.593730594 | 4.128187 | 0.001210965 | 0.208880693 | -0.701972 | CDH3 | mRNA |
| A_23_P49448 | 1.1371297 | 3.434161423 | 3.942695 | 0.001713061 | 0.226017267 | -1.001037 | FA2H | mRNA |
| A_23_P50697 | -1.568295 | 4.340744806 | -3.42883 | 0.004541533 | 0.239667503 | -1.846837 | PSG1 | mRNA |
| A_23_P51085 | 1.5837262 | 3.386518689 | 5.465229 | 0.000111952 | 0.140344925 | 1.3025737 | SPC25 | mRNA |
| A_23_P56494 | 1.4303341 | 4.609364801 | 3.124916 | 0.008129424 | 0.266991578 | -2.353464 | SLC38A11 | mRNA |
| A_23_P56787 | 1.0835507 | 1.926346409 | 4.708336 | 0.000419126 | 0.178530425 | 0.203299 | CNTNAP5 | mRNA |
| A_23_P571 | 1.093568 | 10.25573958 | 2.655111 | 0.019944958 | 0.331869403 | -3.131583 | SLC2A1 | mRNA |
| A_23_P57277 | -1.589912 | 8.339427827 | -5.54066 | 0.0000986 | 0.139893167 | 1.4066357 | C21orf7 | mRNA |
| A_23_P57474 | 1.2786104 | 10.18162931 | 2.297574 | 0.039002306 | 0.409131685 | -3.705745 | OSBP2 | mRNA |
| A_23_P57588 | 1.1838564 | 4.815022213 | 3.939758 | 0.00172254 | 0.226017267 | -1.005804 | GTSE1 | mRNA |
| A_23_P60130 | 1.6886023 | 4.431888944 | 5.493743 | 0.000106684 | 0.140344925 | 1.3420279 | MAL2 | mRNA |
| A_23_P615 | -1.000785 | 3.904096879 | -3.65706 | 0.002939206 | 0.226472045 | -1.468654 | INSRR | mRNA |
| A_23_P61637 | -1.313872 | 7.480848504 | -3.4523 | 0.004342292 | 0.237160825 | -1.807812 | HAL | mRNA |
| A_23_P64121 | 1.6210794 | 2.307823306 | 5.200416 | 0.000176111 | 0.15159608 | 0.9292609 | C11orf41 | mRNA |
| A_23_P64617 | -1.223891 | 3.351818692 | -3.80861 | 0.00220544 | 0.226472045 | -1.219594 | FZD4 | mRNA |
| A_23_P65757 | 1.9804354 | 5.61928767 | 3.462051 | 0.004262167 | 0.237160825 | -1.791613 | CCNB2 | mRNA |
| A_23_P66241 | 1.4566617 | 5.181616224 | 3.624267 | 0.003128273 | 0.226595231 | -1.522784 | MT1M | mRNA |
| A_23_P692 | -1.024433 | 4.563987824 | -3.09211 | 0.008657249 | 0.271135093 | -2.408178 | KCND3 | mRNA |
| A_23_P72668 | -1.482547 | 8.644892251 | -5.17016 | 0.000185582 | 0.15159608 | 0.8858183 | SDPR | mRNA |
| A_23_P76460 | 1.0571794 | 5.185384555 | 3.653292 | 0.002960306 | 0.226472045 | -1.474864 | MYF6 | mRNA |
| A_23_P77043 | -1.100434 | 3.568350676 | -3.31786 | 0.005616188 | 0.248680669 | -2.031642 | CATSPERB | mRNA |
| A_23_P77493 | 1.2108599 | 3.947283575 | 3.38193 | 0.004967827 | 0.24210451 | -1.924894 | TUBB3 | mRNA |
| A_23_P80162 | 1.0028162 | 2.491943638 | 2.356324 | 0.034975402 | 0.397568472 | -3.613132 | TMPRSS3 | mRNA |
| A_23_P88278 | -1.044323 | 5.586998623 | -4.02861 | 0.001458215 | 0.221134227 | -0.862008 | RPGRIP1 | mRNA |
| A_23_P88303 | 1.2078972 | 5.140141083 | 2.778471 | 0.01577464 | 0.310825088 | -2.928921 | HSPA2 | mRNA |
| A_23_P88589 | 1.2834109 | 2.767530918 | 3.973323 | 0.001617346 | 0.226017267 | -0.951379 | NR2F2 | mRNA |
| A_23_P89422 | 1.0413137 | 3.466100639 | 3.375426 | 0.005030046 | 0.242854518 | -1.935724 | ABCA10 | mRNA |
| A_23_P89762 | -1.601518 | 5.094292245 | -6.58528 | 0.0000184 | 0.097591461 | 2.7432434 | PHLPP1 | mRNA |
| A_23_P93141 | -1.776669 | 3.357620331 | -2.45936 | 0.028853484 | 0.376159279 | -3.448885 | GSTA5 | mRNA |
| A_23_P93602 | -1.032771 | 3.024648808 | -3.0695 | 0.009040746 | 0.273473567 | -2.44587 | C6orf58 | mRNA |
| A_23_P94647 | -1.468276 | 5.280033342 | -3.51964 | 0.003818368 | 0.232778983 | -1.696 | OR1L3 | mRNA |
| A_24_P100551 | 1.0466563 | 5.767143858 | 4.398317 | 0.000735331 | 0.198545834 | -0.274342 | SH3RF1 | mRNA |
| A_24_P105733 | -2.243471 | 4.938443369 | -2.87708 | 0.013068019 | 0.29958464 | -2.765844 | TNS1 | mRNA |
| A_24_P120907 | -1.025034 | 2.347733221 | -2.51794 | 0.025846906 | 0.360430244 | -3.354588 | PGM5 | mRNA |
| A_24_P132633 | -1.025021 | 6.741967609 | -3.78168 | 0.00232062 | 0.226472045 | -1.263697 | C9orf71 | mRNA |
| A_24_P160680 | -1.19438 | 2.262634861 | -3.87027 | 0.001963152 | 0.226017267 | -1.118852 | CCDC40 | mRNA |
| A_24_P166397 | -1.041222 | 2.868696949 | -3.06509 | 0.009117367 | 0.273942508 | -2.453208 | KIAA0319 | mRNA |
| A_24_P215240 | -1.818816 | 6.279122579 | -4.78031 | 0.000368471 | 0.175413373 | 0.3120213 | ENKUR | mRNA |
| A_24_P218805 | -1.458522 | 3.583765162 | -3.78412 | 0.002309939 | 0.226472045 | -1.2597 | HOXC10 | mRNA |
| A_24_P220485 | 1.4761836 | 2.020007354 | 5.01175 | 0.000244629 | 0.162553732 | 0.6557915 | OLFML2A | mRNA |
| A_24_P229234 | 1.0464478 | 2.316985042 | 3.207057 | 0.006944806 | 0.257704719 | -2.216435 | MYO16 | mRNA |
| A_24_P238499 | 1.0344367 | 1.917744239 | 4.657617 | 0.000459129 | 0.179866262 | 0.1261836 | C18orf56 | mRNA |
| A_24_P23995 | 1.0446738 | 9.495508153 | 2.807366 | 0.014928958 | 0.307660797 | -2.881221 | RNF187 | mRNA |
| A_24_P24770 | -1.044027 | 1.880118624 | -4.22174 | 0.001017922 | 0.204841282 | -0.552751 | SLC9A10 | mRNA |
| A_24_P250922 | -1.628867 | 7.123529408 | -3.74743 | 0.002476069 | 0.226472045 | -1.319896 | PTGS2 | mRNA |
| A_24_P270460 | 4.4431283 | 9.483552567 | 7.080327 | 0.00000871 | 0.081134697 | 3.3102366 | IFI27 | mRNA |
| A_24_P290692 | 1.1546315 | 3.759473618 | 3.806023 | 0.002216242 | 0.226472045 | -1.223827 | COG6 | mRNA |
| A_24_P296587 | 1.1315758 | 4.507908639 | 2.764397 | 0.0162034 | 0.313873588 | -2.952125 | DLX3 | mRNA |
| A_24_P296698 | 1.2733123 | 10.36857419 | 3.627523 | 0.003108955 | 0.226595231 | -1.517405 | MAP2K3 | mRNA |
| A_24_P297551 | -1.796731 | 3.661453537 | -3.68006 | 0.00281356 | 0.226472045 | -1.430734 | FAM19A2 | mRNA |
| A_24_P309521 | -1.178307 | 3.555242969 | -2.27097 | 0.040966998 | 0.415265921 | -3.747404 | KCNJ5 | mRNA |
| A_24_P31275 | 1.3347509 | 7.080511981 | 4.957983 | 0.000268883 | 0.162928093 | 0.5767326 | ATP1B2 | mRNA |
| A_24_P314786 | -1.511242 | 9.369543425 | -3.68838 | 0.002769461 | 0.226472045 | -1.417025 | SLC4A10 | mRNA |
| A_24_P319736 | -1.536565 | 8.417514916 | -4.74473 | 0.000392668 | 0.177038132 | 0.2583727 | MEIS1 | mRNA |
| A_24_P322354 | 1.2266248 | 3.195122987 | 3.581143 | 0.003395906 | 0.22831998 | -1.594092 | SKA1 | mRNA |
| A_24_P335620 | 1.3653557 | 7.320300463 | 3.262317 | 0.006246784 | 0.252302678 | -2.124252 | SLC7A5 | mRNA |
| A_24_P33895 | -1.02255 | 6.535167362 | -2.69915 | 0.018345099 | 0.325568106 | -3.059425 | ATF3 | mRNA |
| A_24_P349728 | -1.204837 | 3.928008174 | -2.45648 | 0.029009791 | 0.376638305 | -3.453508 | LOC100129312 | mRNA |
| A_24_P365365 | 1.0823676 | 8.255281473 | 4.154967 | 0.001152133 | 0.205935135 | -0.65914 | TCF3 | mRNA |
| A_24_P369232 | -1.17408 | 6.226510338 | -3.74157 | 0.002503708 | 0.226472045 | -1.32952 | CCDC3 | mRNA |
| A_24_P376129 | -1.162777 | 2.510624483 | -4.47637 | 0.000637593 | 0.1889821 | -0.152702 | DFNB31 | mRNA |
| A_24_P377225 | 1.0140039 | 2.668845107 | 2.507541 | 0.026357501 | 0.363500573 | -3.371369 | USP46 | mRNA |
| A_24_P399888 | 1.6333931 | 3.36844467 | 2.599642 | 0.02215427 | 0.342445738 | -3.222097 | CENPM | mRNA |
| A_24_P402825 | -1.213795 | 7.373279556 | -2.50259 | 0.026604032 | 0.364787782 | -3.379353 | CACNA2D3 | mRNA |
| A_24_P410605 | 1.1758483 | 2.807319681 | 5.398828 | 0.000125306 | 0.143794437 | 1.2101338 | ROR1 | mRNA |
| A_24_P413126 | 1.3172842 | 3.578571197 | 2.29915 | 0.038888779 | 0.408998875 | -3.703272 | PMEPA1 | mRNA |
| A_24_P413884 | 1.1425855 | 2.391699592 | 4.220518 | 0.001020228 | 0.204841282 | -0.554693 | CENPA | mRNA |
| A_24_P43144 | 1.3022413 | 2.940950445 | 5.880989 | 0.0000561 | 0.127783614 | 1.8634363 | PDE11A | mRNA |
| A_24_P44780 | -1.288443 | 4.302688516 | -3.02467 | 0.0098519 | 0.27956112 | -2.520563 | LOXHD1 | mRNA |
| A_24_P462899 | 1.0112472 | 5.91040524 | 3.027999 | 0.009789204 | 0.27895134 | -2.515014 | CENPW | mRNA |
| A_24_P49260 | -1.168841 | 5.499010859 | -2.46715 | 0.028434869 | 0.374406941 | -3.436376 | SPTLC3 | mRNA |
| A_24_P54390 | 1.1330997 | 6.475394899 | 2.946963 | 0.01143286 | 0.289657887 | -2.649848 | RASGRP3 | mRNA |
| A_24_P600377 | 1.120576 | 2.230254199 | 3.085151 | 0.00877347 | 0.271258138 | -2.419775 | KIAA1024L | mRNA |
| A_24_P6517 | 1.3616248 | 4.863158763 | 3.317064 | 0.005624719 | 0.248680669 | -2.032963 | PLEKHG1 | mRNA |
| A_24_P77904 | -1.289687 | 2.881719186 | -2.74507 | 0.016810781 | 0.319065491 | -2.983952 | HOXA10 | mRNA |
| A_24_P926507 | 1.7398466 | 8.504440501 | 2.660656 | 0.019736258 | 0.330315445 | -3.122511 | SLC14A1 | mRNA |
| A_24_P930975 | -1.003902 | 7.799205836 | -2.96535 | 0.011037299 | 0.286329907 | -2.619272 | CD300LD | mRNA |
| A_24_P934800 | 1.2497984 | 6.489768452 | 3.135818 | 0.007961257 | 0.266836872 | -2.335281 | ERI2 | mRNA |
| A_24_P94402 | -1.104505 | 1.971153083 | -2.36772 | 0.034241362 | 0.394778134 | -3.59507 | MYCN | mRNA |
| A_32_P125338 | 1.3016771 | 3.758650113 | 2.746852 | 0.016753971 | 0.318611364 | -2.981024 | FAM43B | mRNA |
| A_32_P126375 | -1.556896 | 4.596399872 | -3.89808 | 0.001862983 | 0.226017267 | -1.073553 | NHS | mRNA |
| A_32_P158966 | -1.059794 | 7.66050276 | -2.7409 | 0.016944864 | 0.319594244 | -2.99082 | KLRF1 | mRNA |
| A_32_P178945 | 1.4543872 | 9.128948483 | 2.355956 | 0.034999387 | 0.397568472 | -3.613716 | YOD1 | mRNA |
| A_32_P194312 | 1.0053454 | 3.007215203 | 2.250554 | 0.042538488 | 0.420144349 | -3.779263 | SDK2 | mRNA |
| A_32_P197561 | 1.3234692 | 5.824125153 | 2.887558 | 0.012808885 | 0.296718797 | -2.748473 | EBF1 | mRNA |
| A_32_P356316 | 1.2777674 | 8.1764053 | 3.808408 | 0.002206274 | 0.226472045 | -1.219922 | HLA-DOA | mRNA |
| A_32_P38623 | 1.1839864 | 2.207960292 | 3.671619 | 0.002858999 | 0.226472045 | -1.444638 | PPP1R9A | mRNA |
| A_32_P465742 | 1.2189614 | 6.355833349 | 2.256847 | 0.04204827 | 0.418984603 | -3.769457 | PIP5K1B | mRNA |
| A_32_P65589 | 1.0639449 | 10.19117895 | 3.29214 | 0.00589979 | 0.249862515 | -2.074515 | LOC100130811 | mRNA |
| A_32_P87013 | -1.875659 | 4.169784493 | -3.18133 | 0.007295952 | 0.259102751 | -2.259356 | IL8 | mRNA |
| A_33_P3212109 | 1.3546887 | 3.495446267 | 3.565941 | 0.003495701 | 0.229638557 | -1.619259 | DCDC2 | mRNA |
| A_33_P3213337 | 1.1553393 | 5.232632562 | 3.217331 | 0.006809355 | 0.256602846 | -2.199295 | C15orf53 | mRNA |
| A_33_P3213645 | 1.1903846 | 3.047962293 | 2.978725 | 0.010758299 | 0.285229022 | -2.597035 | ERN2 | mRNA |
| A_33_P3214635 | 1.519717 | 7.721638503 | 2.193161 | 0.047265785 | 0.430566859 | -3.868218 | FECH | mRNA |
| A_33_P3215198 | 1.1113891 | 7.984530009 | 2.275856 | 0.040599521 | 0.413902706 | -3.739771 | CMPK2 | mRNA |
| A_33_P3215953 | -1.25068 | 9.13447711 | -4.58019 | 0.000528006 | 0.185848765 | 0.0076777 | MPZL1 | mRNA |
| A_33_P3216714 | 1.3026203 | 4.523436321 | 2.779703 | 0.015737627 | 0.310697631 | -2.926888 | DNAJC6 | mRNA |
| A_33_P3230269 | -1.083068 | 4.154739731 | -3.37318 | 0.00505174 | 0.242854518 | -1.939469 | GRHL1 | mRNA |
| A_33_P3230548 | 1.5071105 | 4.956609199 | 5.583182 | 0.0000918 | 0.139893167 | 1.4648384 | KIF14 | mRNA |
| A_33_P3231953 | 1.3606815 | 2.588762666 | 3.881896 | 0.00192063 | 0.226017267 | -1.099909 | COL12A1 | mRNA |
| A_33_P3233645 | 2.044263 | 4.837005722 | 4.951707 | 0.000271873 | 0.162928093 | 0.5674727 | MT1G | mRNA |
| A_33_P3236222 | -1.492058 | 5.856096806 | -2.77376 | 0.015916766 | 0.311375356 | -2.936683 | LOC391766 | mRNA |
| A_33_P3237150 | 1.0667942 | 2.424932927 | 3.056745 | 0.009264453 | 0.274889306 | -2.467122 | BMP2 | mRNA |
| A_33_P3237215 | -1.039473 | 3.96973405 | -2.71732 | 0.017722498 | 0.323272265 | -3.029601 | FBXO24 | mRNA |
| A_33_P3237482 | 1.4445 | 4.589730428 | 3.18348 | 0.007265939 | 0.259082821 | -2.255769 | KIF6 | mRNA |
| A_33_P3239282 | 1.1217026 | 4.356546922 | 2.750736 | 0.016630541 | 0.3178098 | -2.97463 | KCNA2 | mRNA |
| A_33_P3239347 | -1.207752 | 5.967587473 | -2.96825 | 0.010976293 | 0.286329907 | -2.614458 | NKX3-1 | mRNA |
| A_33_P3243093 | 1.1107241 | 3.45411654 | 2.797768 | 0.015204792 | 0.308763195 | -2.897074 | RGS5 | mRNA |
| A_33_P3244007 | 1.1710928 | 4.018659038 | 4.26681 | 0.000936536 | 0.203593727 | -0.481277 | NEBL | mRNA |
| A_33_P3244543 | -1.776527 | 3.236912262 | -4.22076 | 0.001019779 | 0.204841282 | -0.554315 | C5orf48 | mRNA |
| A_33_P3245799 | -1.065642 | 7.927485295 | -2.70966 | 0.01798236 | 0.324165891 | -3.042177 | GPR114 | mRNA |
| A_33_P3245824 | 1.0508702 | 9.356853811 | 2.718078 | 0.017696884 | 0.323272265 | -3.028351 | PQLC1 | mRNA |
| A_33_P3252974 | -1.123925 | 4.739498005 | -6.52491 | 0.0000201 | 0.097591461 | 2.6712436 | SCRT2 | mRNA |
| A_33_P3253596 | 1.5975205 | 4.589570272 | 3.883176 | 0.001916008 | 0.226017267 | -1.097824 | KIF4A | mRNA |
| A_33_P3256680 | 1.0400985 | 5.993785879 | 3.652734 | 0.002963445 | 0.226472045 | -1.475784 | MFHAS1 | mRNA |
| A_33_P3258206 | -1.012976 | 4.507495476 | -2.28741 | 0.039742029 | 0.411283394 | -3.721679 | OR6N2 | mRNA |
| A_33_P3263666 | 1.6021963 | 8.572264922 | 2.360892 | 0.034679481 | 0.396707264 | -3.605898 | ANKRD9 | mRNA |
| A_33_P3268783 | -1.005912 | 5.904834717 | -3.44492 | 0.004404029 | 0.237399693 | -1.820092 | AMZ1 | mRNA |
| A_33_P3269403 | 1.1917292 | 5.531760097 | 4.668672 | 0.000450084 | 0.178530425 | 0.1430263 | FOXI1 | mRNA |
| A_33_P3273767 | 1.1009406 | 3.421633771 | 5.15637 | 0.000190072 | 0.152092253 | 0.8659699 | GJB7 | mRNA |
| A_33_P3276845 | -1.390884 | 5.542532814 | -3.39226 | 0.004870612 | 0.24210451 | -1.907698 | LOC100288018 | mRNA |
| A_33_P3278275 | -1.106344 | 5.160796152 | -5.43644 | 0.000117548 | 0.14260695 | 1.2625949 | TXNDC8 | mRNA |
| A_33_P3279515 | -1.59579 | 7.249520436 | -6.05206 | 0.0000425 | 0.1141012 | 2.0852175 | LOC729451 | mRNA |
| A_33_P3280521 | -1.205014 | 6.089895665 | -3.79672 | 0.002255541 | 0.226472045 | -1.239052 | MFAP3L | mRNA |
| A_33_P3282325 | 1.103404 | 3.263004694 | 3.478572 | 0.004129757 | 0.237120028 | -1.764166 | DAZ1 | mRNA |
| A_33_P3285764 | -1.026391 | 8.843556058 | -2.6347 | 0.020731718 | 0.33535003 | -3.164937 | EPHB1 | mRNA |
| A_33_P3287646 | 1.373859 | 9.697115131 | 3.533237 | 0.003720589 | 0.231756767 | -1.673448 | HSPB1 | mRNA |
| A_33_P3289661 | -1.106846 | 8.636571152 | -4.2985 | 0.000883353 | 0.203593727 | -0.431185 | OCR1 | mRNA |
| A_33_P3294277 | -1.017815 | 8.36479262 | -2.47974 | 0.027770952 | 0.371350704 | -3.416144 | CYP4F3 | mRNA |
| A_33_P3295029 | 1.0990869 | 3.188544123 | 3.014516 | 0.010045408 | 0.281012432 | -2.537467 | KBTBD12 | mRNA |
| A_33_P3296499 | -1.072437 | 7.219117071 | -3.41676 | 0.00464761 | 0.241255554 | -1.866923 | PTPRK | mRNA |
| A_33_P3299254 | 1.2158343 | 8.087333912 | 3.124509 | 0.00813578 | 0.266991578 | -2.354144 | VPREB3 | mRNA |
| A_33_P3299761 | -1.040276 | 9.343572927 | -2.3788 | 0.03354238 | 0.392800431 | -3.577496 | KRT23 | mRNA |
| A_33_P3300837 | -1.335966 | 10.32129402 | -2.33588 | 0.036329387 | 0.402205993 | -3.645447 | LDB2 | mRNA |
| A_33_P3301351 | -1.127179 | 2.529440716 | -3.55203 | 0.003589628 | 0.229983881 | -1.642302 | RBM44 | mRNA |
| A_33_P3302632 | 1.4152881 | 9.369429512 | 7.53942 | 0.00000449 | 0.081134697 | 3.7999614 | HIST1H2BE | mRNA |
| A_33_P3305835 | 1.2283598 | 2.324034824 | 2.357728 | 0.034884233 | 0.39725774 | -3.61091 | VPREB1 | mRNA |
| A_33_P3306252 | -1.65966 | 3.035652457 | -2.77392 | 0.015912106 | 0.311375356 | -2.936429 | FLJ34223 | mRNA |
| A_33_P3312217 | -1.013889 | 6.461499399 | -3.57673 | 0.003424604 | 0.229330807 | -1.601404 | LOC729706 | mRNA |
| A_33_P3314176 | 1.2290105 | 10.92818349 | 2.637519 | 0.020621304 | 0.335261027 | -3.160334 | FAM46C | mRNA |
| A_33_P3319022 | -1.450769 | 5.485286191 | -2.74325 | 0.016869275 | 0.319186414 | -2.986955 | CLEC9A | mRNA |
| A_33_P3319810 | -1.342344 | 7.37817275 | -2.46599 | 0.028496834 | 0.374982761 | -3.438239 | FAM123C | mRNA |
| A_33_P3320082 | 1.2735386 | 2.275393359 | 3.89583 | 0.001870891 | 0.226017267 | -1.077216 | NFIB | mRNA |
| A_33_P3321293 | 1.1197418 | 2.981012167 | 2.238019 | 0.043531264 | 0.422283448 | -3.798768 | IQGAP3 | mRNA |
| A_33_P3326210 | 1.3218822 | 7.526384956 | 2.566327 | 0.023593688 | 0.350034611 | -3.276249 | ESCO2 | mRNA |
| A_33_P3335506 | 1.1128216 | 4.827105381 | 2.170588 | 0.04925712 | 0.435831657 | -3.902954 | FCRL5 | mRNA |
| A_33_P3336113 | -1.531306 | 5.744867235 | -4.15973 | 0.001141989 | 0.205935135 | -0.651537 | TIGD3 | mRNA |
| A_33_P3346891 | -1.006651 | 9.478323483 | -3.11925 | 0.008218264 | 0.267644214 | -2.362918 | MYBL1 | mRNA |
| A_33_P3347343 | -1.205937 | 5.142446014 | -2.7152 | 0.017794121 | 0.323834576 | -3.033086 | CCDC102B | mRNA |
| A_33_P3348594 | 1.052679 | 1.887669097 | 5.417317 | 0.000121428 | 0.142709888 | 1.235953 | OR5R1 | mRNA |
| A_33_P3350488 | 1.0282759 | 5.284652131 | 3.781228 | 0.002322618 | 0.226472045 | -1.264443 | NUSAP1 | mRNA |
| A_33_P3353210 | -1.337749 | 4.919094257 | -3.79161 | 0.002277474 | 0.226472045 | -1.247436 | CADM2 | mRNA |
| A_33_P3356320 | 1.3940932 | 5.385985221 | 3.001808 | 0.010292987 | 0.282091827 | -2.558623 | C17orf97 | mRNA |
| A_33_P3358342 | 1.0611427 | 10.69789055 | 2.197643 | 0.046879468 | 0.429374593 | -3.861303 | SIAH2 | mRNA |
| A_33_P3360611 | 1.3211583 | 2.633082311 | 3.354699 | 0.005233617 | 0.243331186 | -1.970247 | MPP2 | mRNA |
| A_33_P3362641 | 1.1943212 | 7.598694957 | 2.523274 | 0.025588595 | 0.359988302 | -3.345969 | CISD2 | mRNA |
| A_33_P3363637 | 1.2352027 | 6.161539097 | 2.266509 | 0.041305889 | 0.416754538 | -3.75438 | BLNK | mRNA |
| A_33_P3365760 | 1.0544091 | 8.573283852 | 3.223115 | 0.006734262 | 0.256602846 | -2.189644 | STAP1 | mRNA |
| A_33_P3366589 | 1.1498949 | 2.377462228 | 3.628187 | 0.003105032 | 0.226595231 | -1.516308 | OR56B4 | mRNA |
| A_33_P3367701 | -1.053275 | 7.769342642 | -3.09496 | 0.00861009 | 0.271135093 | -2.403428 | TMEM164 | mRNA |
| A_33_P3376116 | 1.1318216 | 2.94233104 | 2.535842 | 0.024989972 | 0.357765529 | -3.325648 | SPC24 | mRNA |
| A_33_P3377529 | -1.416623 | 2.641338418 | -3.69398 | 0.002740148 | 0.226472045 | -1.407792 | HOXA4 | mRNA |
| A_33_P3378680 | -1.139066 | 6.219436021 | -3.25972 | 0.00627791 | 0.252302678 | -2.128577 | ARHGAP26 | mRNA |
| A_33_P3379341 | 4.3153936 | 4.281564049 | 12.03138 | 2.23E-08 | 0.001679445 | 7.1489121 | LOC391322 | mRNA |
| A_33_P3380642 | 1.4254189 | 5.172402602 | 3.666724 | 0.002885706 | 0.226472045 | -1.452708 | FRAS1 | mRNA |
| A_33_P3382493 | -1.55148 | 8.714387914 | -2.33071 | 0.036679723 | 0.40279851 | -3.653606 | SIGLEC14 | mRNA |
| A_33_P3383551 | -1.248756 | 11.1032226 | -2.23067 | 0.044123679 | 0.422881147 | -3.810188 | SEC14L3 | mRNA |
| A_33_P3384452 | 1.1256192 | 10.03074611 | 3.147455 | 0.007785575 | 0.265581675 | -2.315868 | TFDP1 | mRNA |
| A_33_P3387420 | 1.8286523 | 3.059887532 | 3.748609 | 0.002470547 | 0.226472045 | -1.31796 | PKHD1 | mRNA |
| A_33_P3388806 | 1.674349 | 2.558086726 | 3.603101 | 0.003256842 | 0.226595231 | -1.557767 | FPGT-TNNI3K | mRNA |
| A_33_P3390172 | 1.0040477 | 2.025281069 | 3.080163 | 0.008857775 | 0.271775478 | -2.428091 | ADAMDEC1 | mRNA |
| A_33_P3393801 | -1.101232 | 12.125134 | -2.24566 | 0.042923208 | 0.420603819 | -3.786877 | PDZK1IP1 | mRNA |
| A_33_P3395083 | 1.1309892 | 2.569619775 | 4.496478 | 0.000614663 | 0.1889821 | -0.121512 | ACSM4 | mRNA |
| A_33_P3395532 | 1.3203305 | 8.776026521 | 3.294597 | 0.005872087 | 0.249761539 | -2.070419 | IFIT1B | mRNA |
| A_33_P3397865 | 1.3894814 | 6.34504889 | 3.478996 | 0.004126411 | 0.237120028 | -1.763461 | TNNT1 | mRNA |
| A_33_P3399433 | 1.0871084 | 9.044067522 | 3.532163 | 0.003728222 | 0.231756767 | -1.67523 | C20orf27 | mRNA |
| A_33_P3402615 | 1.7748545 | 5.744150205 | 2.888831 | 0.012777744 | 0.296649009 | -2.746362 | SLC6A9 | mRNA |
| A_33_P3406171 | 1.0284308 | 2.57133774 | 4.319105 | 0.000850458 | 0.202947597 | -0.398694 | PABPC5 | mRNA |
| A_33_P3406567 | 1.035403 | 7.713598894 | 2.512694 | 0.026103254 | 0.361585355 | -3.363055 | MS4A1 | mRNA |
| A_33_P3406677 | 1.1914102 | 3.02791502 | 3.067383 | 0.00907746 | 0.273473567 | -2.449394 | ADH7 | mRNA |
| A_33_P3413468 | 1.2038466 | 1.737678871 | 3.860428 | 0.001999931 | 0.226017267 | -1.134913 | EDA2R | mRNA |
| A_33_P3416966 | 1.0188906 | 2.58414697 | 2.51421 | 0.026028906 | 0.3611707 | -3.360608 | C6orf168 | mRNA |
| A_33_P3424612 | 1.0464224 | 9.15533193 | 2.331529 | 0.036624133 | 0.40279851 | -3.652317 | LOC100653210 | mRNA |
| A_33_P3482534 | 1.1669202 | 6.047734033 | 2.482312 | 0.027637345 | 0.371251196 | -3.412012 | LOC613266 | mRNA |
| A_33_P3493097 | 1.0475253 | 2.562409984 | 2.554573 | 0.024122842 | 0.352256158 | -3.295312 | LOC648149 | mRNA |
| A_33_P3692984 | 1.4606925 | 2.985128512 | 5.059401 | 0.000225042 | 0.158611722 | 0.7254421 | LOC283486 | mRNA |
| A_33_P3807062 | 1.3974049 | 3.534393403 | 4.801094 | 0.000355063 | 0.174988778 | 0.3432562 | HJURP | mRNA |
| A_33_P3815064 | -1.085841 | 5.681666013 | -2.57534 | 0.023195717 | 0.348096351 | -3.261621 | LOC283575 | mRNA |
| A_33_P3841368 | 1.0704899 | 6.849995243 | 2.654697 | 0.01996061 | 0.331869403 | -3.13226 | LOC286161 | mRNA |
| A_33_P3857217 | 1.0877562 | 3.349017017 | 3.066738 | 0.00908869 | 0.273473567 | -2.450469 | LOC286189 | mRNA |
| A_33_P3865368 | -1.010895 | 9.460944425 | -3.18198 | 0.007286926 | 0.259082821 | -2.258279 | LOC254896 | mRNA |
| p10044 | 1.3694698 | 2.774127106 | 3.791847 | 0.002276435 | 0.226472045 | -1.24704 | ENSG00000226674 | lncRNA |
| p10191 | 1.00618 | 2.916417786 | 3.035274 | 0.009653673 | 0.278174499 | -2.502897 | ENSG00000260142 | lncRNA |
| p10205 | 1.0110202 | 2.980305036 | 2.982825 | 0.010674137 | 0.28423542 | -2.590213 | ENSG00000225953 | lncRNA |
| p10295 | 1.1282594 | 3.42344017 | 3.786696 | 0.002298721 | 0.226472045 | -1.255481 | ENSG00000226442 | lncRNA |
| p10365 | -1.052675 | 3.390660874 | -2.36435 | 0.034456973 | 0.395928829 | -3.600416 | ENSG00000233993 | lncRNA |
| p10421 | -1.513444 | 4.449564799 | -3.59069 | 0.003334744 | 0.226595231 | -1.578302 | ENSG00000126005 | lncRNA |
| p10452 | 1.1005668 | 2.294534032 | 3.840545 | 0.002076365 | 0.226017267 | -1.167374 | ENSG00000233376 | lncRNA |
| p1046 | 1.0063777 | 3.991814028 | 4.749009 | 0.00038967 | 0.177038132 | 0.264841 | ENSG00000230817 | lncRNA |
| p10475 | 1.1369878 | 2.524163628 | 3.933991 | 0.001741305 | 0.226017267 | -1.015166 | ENSG00000225563 | lncRNA |
| p10592 | -1.205672 | 6.464965555 | -2.80112 | 0.015107833 | 0.308034284 | -2.891535 | ENSG00000255438 | lncRNA |
| p10762 | -1.312168 | 3.162100298 | -2.66662 | 0.019514215 | 0.329735837 | -3.112751 | ENSG00000233754 | lncRNA |
| p10847 | -1.173664 | 3.045390536 | -2.36657 | 0.034315143 | 0.395265256 | -3.596903 | ENSG00000215533 | lncRNA |
| p11043 | 1.2781737 | 7.499170823 | 2.972792 | 0.010881221 | 0.286095806 | -2.606903 | ENSG00000260655 | lncRNA |
| p11338 | -1.645429 | 4.036224273 | -4.43829 | 0.000683482 | 0.191826429 | -0.211941 | ENSG00000235257 | lncRNA |
| p11461 | 1.0293781 | 1.947582245 | 3.981859 | 0.001591661 | 0.225358229 | -0.937559 | ENSG00000241469 | lncRNA |
| p11510 | -1.40085 | 2.885578552 | -4.48351 | 0.000629352 | 0.1889821 | -0.141622 | ENSG00000249993 | lncRNA |
| p116 | -1.017766 | 3.699117033 | -2.45035 | 0.02934506 | 0.377521574 | -3.463339 | ENSG00000233478 | lncRNA |
| p11992 | 1.0797836 | 3.267335373 | 3.639041 | 0.003041599 | 0.226595231 | -1.498385 | ENSG00000241570 | lncRNA |
| p12061 | -1.099448 | 3.170036483 | -2.8449 | 0.013896814 | 0.302942828 | -2.819154 | ENSG00000240207 | lncRNA |
| p12107 | 1.8283228 | 2.283637043 | 4.01502 | 0.001495785 | 0.222884089 | -0.883948 | ENSG00000244302 | lncRNA |
| p12154 | 1.3103912 | 2.976207417 | 3.099314 | 0.008538438 | 0.271135093 | -2.39616 | ENSG00000228952 | lncRNA |
| p12335 | -1.042223 | 2.102010947 | -3.2116 | 0.006884563 | 0.257290943 | -2.208853 | ENSG00000260265 | lncRNA |
| p12341 | -1.180901 | 6.552117559 | -3.69218 | 0.002749532 | 0.226472045 | -1.410758 | ENSG00000249036 | lncRNA |
| p12500 | 1.299632 | 2.685846935 | 2.547164 | 0.0244623 | 0.354553964 | -3.307319 | ENSG00000234828 | lncRNA |
| p12637 | 1.2588849 | 2.861548127 | 3.230837 | 0.006635306 | 0.25593825 | -2.176762 | ENSG00000263327 | lncRNA |
| p12686 | -1.206433 | 2.861833785 | -3.02014 | 0.009937773 | 0.280506532 | -2.528105 | ENSG00000255458 | lncRNA |
| p1273 | -1.280334 | 4.719177505 | -4.27829 | 0.000916895 | 0.203593727 | -0.463112 | ENSG00000224977 | lncRNA |
| p12757 | 1.0090414 | 4.898848977 | 2.441353 | 0.029843564 | 0.37899564 | -3.477746 | ENSG00000250877 | lncRNA |
| p12771 | -1.014403 | 5.405280431 | -2.16631 | 0.049643666 | 0.436526493 | -3.909527 | ENSG00000251442 | lncRNA |
| p12776 | -1.747208 | 4.486053178 | -3.44891 | 0.004370562 | 0.23738443 | -1.813457 | ENSG00000249307 | lncRNA |
| p12864 | 1.074657 | 3.917101675 | 3.737335 | 0.002523882 | 0.226472045 | -1.336479 | ENSG00000249464 | lncRNA |
| p12974 | 1.3507101 | 2.729581687 | 5.562748 | 0.000095 | 0.139893167 | 1.4369069 | ENSG00000248431 | lncRNA |
| p13038 | 1.0284827 | 5.061470574 | 3.686113 | 0.002781387 | 0.226472045 | -1.420753 | ENSG00000248925 | lncRNA |
| p13190 | 1.0310947 | 3.734905004 | 3.462735 | 0.004256604 | 0.237160825 | -1.790477 | ENSG00000248285 | lncRNA |
| p13233 | -1.661354 | 3.111417004 | -3.19214 | 0.007146278 | 0.259037125 | -2.24132 | ENSG00000250348 | lncRNA |
| p13285 | -1.299641 | 4.581719654 | -2.19629 | 0.046995947 | 0.429465169 | -3.863394 | ENSG00000251093 | lncRNA |
| p13287 | -1.022111 | 9.211724509 | -2.81707 | 0.014655017 | 0.306707396 | -2.865181 | ENSG00000248323 | lncRNA |
| p13427 | 1.0254398 | 3.072419956 | 3.111012 | 0.008349063 | 0.269394998 | -2.376653 | ENSG00000245146 | lncRNA |
| p13564 | -1.01231 | 2.240087392 | -3.1777 | 0.00734693 | 0.259658888 | -2.265414 | ENSG00000249326 | lncRNA |
| p13565 | 1.0494143 | 3.320795882 | 3.036676 | 0.009627759 | 0.277947809 | -2.50056 | ENSG00000248994 | lncRNA |
| p13577 | 1.0641191 | 2.853156811 | 3.62702 | 0.003111932 | 0.226595231 | -1.518236 | ENSG00000215231 | lncRNA |
| p13619 | -2.063425 | 3.85302265 | -6.18061 | 0.0000346 | 0.11060151 | 2.2484387 | ENSG00000249662 | lncRNA |
| p1365 | 1.1534201 | 4.066355653 | 3.536307 | 0.003698872 | 0.231756767 | -1.66836 | ENSG00000261252 | lncRNA |
| p13663 | -1.215145 | 3.517126581 | -5.06624 | 0.000222369 | 0.158611722 | 0.7354044 | ENSG00000261604 | lncRNA |
| p13690 | -1.153662 | 2.175293317 | -4.16139 | 0.001138474 | 0.205935135 | -0.648887 | ENSG00000247345 | lncRNA |
| p13856 | -1.845552 | 5.875004861 | -3.19758 | 0.007072174 | 0.258477989 | -2.232249 | ENSG00000229855 | lncRNA |
| p13862 | 1.0469633 | 2.635761232 | 3.747817 | 0.002474257 | 0.226472045 | -1.319261 | ENSG00000250438 | lncRNA |
| p13863 | 1.0282729 | 3.607666406 | 3.262233 | 0.00624778 | 0.252302678 | -2.12439 | ENSG00000249112 | lncRNA |
| p13974 | 1.0412864 | 3.216248107 | 2.233035 | 0.043931997 | 0.422754419 | -3.806511 | ENSG00000245812 | lncRNA |
| p14007 | 1.0128887 | 3.372327457 | 5.200836 | 0.000175983 | 0.15159608 | 0.9298641 | ENSG00000248222 | lncRNA |
| p14102 | 1.2362722 | 5.143232194 | 4.376851 | 0.00076484 | 0.199930261 | -0.307951 | ENSG00000183674 | lncRNA |
| p1423 | -1.089044 | 2.144833246 | -3.44737 | 0.004383465 | 0.23738443 | -1.816021 | ENSG00000228058 | lncRNA |
| p14236 | -1.106418 | 8.653957189 | -3.20116 | 0.007023712 | 0.258164555 | -2.226266 | ENSG00000232295 | lncRNA |
| p14270 | -3.480313 | 3.476748613 | -2.95658 | 0.011224328 | 0.288471389 | -2.633864 | ENSG00000234426 | lncRNA |
| p14273 | 1.1850668 | 2.860388459 | 4.112934 | 0.001245845 | 0.208880693 | -0.726407 | ENSG00000224605 | lncRNA |
| p14353 | -1.259395 | 8.285047112 | -3.40448 | 0.004758076 | 0.241999872 | -1.887359 | ENSG00000229017 | lncRNA |
| p14378 | 1.9166371 | 3.35224402 | 3.320993 | 0.005582548 | 0.248656143 | -2.026414 | ENSG00000226599 | lncRNA |
| p14396 | -1.000085 | 3.35617184 | -3.69157 | 0.002752706 | 0.226472045 | -1.411759 | ENSG00000226032 | lncRNA |
| p14421 | 1.0722969 | 3.067177828 | 3.162519 | 0.007563918 | 0.262182135 | -2.290739 | ENSG00000231720 | lncRNA |
| p14473 | 1.0604277 | 2.416778946 | 2.25856 | 0.041915714 | 0.41880636 | -3.766786 | ENSG00000229401 | lncRNA |
| p14508 | -1.221035 | 2.629765635 | -3.25884 | 0.00628849 | 0.252302678 | -2.130043 | ENSG00000235781 | lncRNA |
| p14597 | 1.3391057 | 3.516177894 | 4.264228 | 0.000941013 | 0.203612481 | -0.485365 | ENSG00000229655 | lncRNA |
| p14679 | -1.235735 | 2.843119763 | -5.05614 | 0.00022633 | 0.158611722 | 0.7206851 | ENSG00000228624 | lncRNA |
| p14691 | -1.131506 | 5.717341122 | -4.11248 | 0.001246891 | 0.208880693 | -0.727129 | ENSG00000234117 | lncRNA |
| p14756 | -1.122732 | 2.687985022 | -4.0498 | 0.001401577 | 0.220182011 | -0.827853 | ENSG00000227748 | lncRNA |
| p14765 | 1.1796048 | 5.034675857 | 2.826231 | 0.014401059 | 0.305759817 | -2.850039 | ENSG00000232891 | lncRNA |
| p14783 | 1.1321234 | 2.611399482 | 3.88788 | 0.001899107 | 0.226017267 | -1.090161 | ENSG00000231863 | lncRNA |
| p14852 | 1.2971854 | 2.588301228 | 3.368002 | 0.005102022 | 0.242854518 | -1.948087 | ENSG00000238033 | lncRNA |
| p14910 | -1.17257 | 4.312712923 | -2.92362 | 0.011955191 | 0.291730427 | -2.688628 | ENSG00000229679 | lncRNA |
| p15094 | -1.189693 | 4.080066661 | -2.21098 | 0.0457477 | 0.427055301 | -3.840698 | ENSG00000240790 | lncRNA |
| p15209 | -1.212562 | 3.360983892 | -4.85442 | 0.000322932 | 0.172174797 | 0.4230693 | ENSG00000241269 | lncRNA |
| p15270 | -1.370618 | 3.499071836 | -3.76879 | 0.002377914 | 0.226472045 | -1.284833 | ENSG00000214870 | lncRNA |
| p15277 | -1.212848 | 3.006976163 | -3.82021 | 0.00215762 | 0.226472045 | -1.20061 | ENSG00000253552 | lncRNA |
| p1532 | -1.279914 | 2.83386809 | -3.40516 | 0.004751875 | 0.241999872 | -1.886225 | ENSG00000233968 | lncRNA |
| p15351 | -1.123033 | 2.845709197 | -2.43349 | 0.030285925 | 0.380360579 | -3.490325 | ENSG00000233977 | lncRNA |
| p15461 | 1.1421796 | 1.974257161 | 4.192342 | 0.001074906 | 0.204841282 | -0.599518 | ENSG00000224897 | lncRNA |
| p15609 | 1.145313 | 2.876620625 | 3.926302 | 0.001766654 | 0.226017267 | -1.027656 | ENSG00000253496 | lncRNA |
| p15783 | 1.097054 | 3.26995722 | 6.822923 | 0.0000128 | 0.081134697 | 3.020589 | ENSG00000253661 | lncRNA |
| p16076 | -1.68582 | 7.398871209 | -3.23259 | 0.006613098 | 0.25593825 | -2.173844 | ENSG00000246130 | lncRNA |
| p16079 | -1.10766 | 7.842269193 | -2.5139 | 0.026043972 | 0.361229841 | -3.361105 | ENSG00000246582 | lncRNA |
| p16164 | 1.0214045 | 5.818196697 | 2.214875 | 0.045421848 | 0.425890694 | -3.834668 | ENSG00000253924 | lncRNA |
| p1619 | 1.1716917 | 2.92536185 | 4.558773 | 0.000548898 | 0.187614587 | -0.025278 | ENSG00000227877 | lncRNA |
| p16286 | 1.1074405 | 4.66093699 | 4.313174 | 0.000859796 | 0.202947597 | -0.408041 | ENSG00000253197 | lncRNA |
| p16504 | -1.101467 | 9.174464503 | -2.68652 | 0.018790693 | 0.327778185 | -3.080146 | ENSG00000234506 | lncRNA |
| p16607 | 1.4256999 | 2.604720673 | 3.714425 | 0.00263591 | 0.226472045 | -1.374145 | ENSG00000244757 | lncRNA |
| p16726 | -1.002136 | 5.292431589 | -2.82221 | 0.014512069 | 0.306211164 | -2.856691 | ENSG00000254396 | lncRNA |
| p16742 | -1.000384 | 4.606563938 | -3.46212 | 0.004261599 | 0.237160825 | -1.791497 | ENSG00000230729 | lncRNA |
| p16750 | 1.2494252 | 5.658231266 | 4.524317 | 0.000584318 | 0.188825047 | -0.078433 | ENSG00000235387 | lncRNA |
| p16753 | 1.1711122 | 6.182392364 | 3.184756 | 0.007248191 | 0.259082821 | -2.253641 | ENSG00000250850 | lncRNA |
| p16909 | 1.7453912 | 3.30671311 | 5.175596 | 0.000183841 | 0.15159608 | 0.8936392 | ENSG00000270102 | lncRNA |
| p16985 | -1.562536 | 3.694625917 | -4.67114 | 0.000448086 | 0.178530425 | 0.1467919 | ENSG00000230020 | lncRNA |
| p17079 | -2.115392 | 3.743052824 | -4.33678 | 0.000823243 | 0.201045137 | -0.370864 | ENSG00000229702 | lncRNA |
| p17152 | 1.2300364 | 2.297927721 | 3.629772 | 0.003095686 | 0.226595231 | -1.51369 | ENSG00000236836 | lncRNA |
| p1724 | -1.057893 | 7.237568039 | -2.3235 | 0.037173814 | 0.403930901 | -3.664978 | ENSG00000231233 | lncRNA |
| p1948 | -1.169459 | 3.109817332 | -2.7376 | 0.017051581 | 0.320161949 | -2.996248 | ENSG00000234736 | lncRNA |
| p1960 | 1.0420075 | 2.957588351 | 2.604786 | 0.021939766 | 0.340959369 | -3.213722 | ENSG00000228527 | lncRNA |
| p1965 | 1.1702559 | 4.906264455 | 2.915507 | 0.012142197 | 0.292868038 | -2.702098 | ENSG00000254271 | lncRNA |
| p2046 | -1.209814 | 2.839229249 | -3.01269 | 0.01008054 | 0.281012432 | -2.540501 | ENSG00000230962 | lncRNA |
| p2169 | 1.4065902 | 2.756583917 | 3.236225 | 0.006567136 | 0.255276642 | -2.167775 | ENSG00000251364 | lncRNA |
| p2260 | 1.6703567 | 3.319030722 | 4.573924 | 0.000534035 | 0.1859471 | -0.001962 | ENSG00000255109 | lncRNA |
| p2301 | 1.2915345 | 6.002864639 | 3.766185 | 0.002389683 | 0.226472045 | -1.289112 | ENSG00000255240 | lncRNA |
| p2441 | 1.0867798 | 5.2329068 | 4.002492 | 0.001531291 | 0.22408385 | -0.904187 | ENSG00000254698 | lncRNA |
| p2455 | -1.015544 | 2.652143736 | -3.0447 | 0.009480808 | 0.276745 | -2.48719 | ENSG00000255471 | lncRNA |
| p2495 | 1.3161091 | 4.473124049 | 5.76946 | 0.0000673 | 0.132760966 | 1.7160246 | ENSG00000255484 | lncRNA |
| p2544 | 1.0423753 | 2.049656516 | 3.235482 | 0.006576489 | 0.255376244 | -2.169013 | ENSG00000255248 | lncRNA |
| p2567 | 1.2752539 | 4.730993934 | 3.840762 | 0.002075515 | 0.226017267 | -1.167019 | ENSG00000260209 | lncRNA |
| p2639 | -1.013773 | 8.799237074 | -3.08036 | 0.0088544 | 0.271775478 | -2.42776 | ENSG00000254665 | lncRNA |
| p2648 | -1.000531 | 4.317664183 | -2.48923 | 0.027280647 | 0.369867205 | -3.400882 | ENSG00000254865 | lncRNA |
| p28976 | -1.004104 | 2.762042404 | -3.62294 | 0.003136154 | 0.226595231 | -1.524969 | ENSG00000267272 | lncRNA |
| p29072 | -1.142294 | 2.740001586 | -3.03311 | 0.00969371 | 0.278174499 | -2.506494 | ENSG00000258168 | lncRNA |
| p29122 | -1.037815 | 8.207910091 | -4.35822 | 0.000791444 | 0.199930261 | -0.337175 | ENSG00000235370 | lncRNA |
| p29245 | -1.175104 | 4.544327634 | -5.33913 | 0.000138739 | 0.150068671 | 1.126357 | ENSG00000179818 | lncRNA |
| p29407 | -1.006552 | 5.351726087 | -4.12651 | 0.00121476 | 0.208880693 | -0.704664 | ENSG00000249082 | lncRNA |
| p2999 | 1.1522481 | 5.755285811 | 4.078168 | 0.001329266 | 0.213184189 | -0.782211 | ENSG00000255087 | lncRNA |
| p3067 | -1.297197 | 7.751137392 | -2.52165 | 0.025666852 | 0.359988302 | -3.34859 | ENSG00000255801 | lncRNA |
| p3071 | -1.08854 | 5.358885016 | -2.19654 | 0.046974353 | 0.429465169 | -3.863007 | ENSG00000214851 | lncRNA |
| p3160 | -1.380067 | 3.871982229 | -4.70609 | 0.000420821 | 0.178530425 | 0.1998867 | ENSG00000258101 | lncRNA |
| p3269 | 1.4551648 | 3.085586393 | 3.108179 | 0.008394537 | 0.269603695 | -2.381377 | ENSG00000247131 | lncRNA |
| p3320 | -1.12765 | 3.594277283 | -2.27798 | 0.040440911 | 0.413631218 | -3.736454 | ENSG00000246363 | lncRNA |
| p3321 | -1.137535 | 3.849585606 | -2.43492 | 0.030204801 | 0.380293039 | -3.488032 | ENSG00000257156 | lncRNA |
| p33492 | -1.008286 | 6.478350498 | -2.52514 | 0.025498974 | 0.359455349 | -3.342958 | ENSG00000186526 | lncRNA |
| p34576_v4 | -1.26424 | 3.181863702 | -2.51092 | 0.026190368 | 0.362324986 | -3.365913 | ENSG00000267506 | lncRNA |
| p34643_v4 | -1.021775 | 2.487898444 | -3.1917 | 0.007152349 | 0.259037125 | -2.242059 | ENSG00000267339 | lncRNA |
| p35018_v4 | -1.473613 | 3.974711045 | -4.24641 | 0.000972513 | 0.204841282 | -0.513598 | ENSG00000223783 | lncRNA |
| p35145_v4 | 1.569731 | 2.944174319 | 5.687427 | 0.0000771 | 0.132760966 | 1.6061733 | ENSG00000250708 | lncRNA |
| p35184_v4 | -1.13972 | 3.408166587 | -2.63595 | 0.020682606 | 0.335261027 | -3.162893 | ENSG00000250802 | lncRNA |
| p35248_v4 | -1.052616 | 3.581885961 | -5.62875 | 0.000085 | 0.139060509 | 1.5268637 | ENSG00000234206 | lncRNA |
| p35437_v4 | -1.597547 | 2.599616201 | -3.47667 | 0.004144762 | 0.237120028 | -1.76732 | ENSG00000254101 | lncRNA |
| p35453_v4 | 1.4777165 | 3.825683732 | 4.347338 | 0.000807425 | 0.199930261 | -0.354269 | ENSG00000224935 | lncRNA |
| p3636 | 1.2860419 | 1.917112104 | 2.925961 | 0.011901748 | 0.291727186 | -2.684739 | ENSG00000257164 | lncRNA |
| p36520_v4 | -1.112191 | 8.435473402 | -3.72551 | 0.002581095 | 0.226472045 | -1.355918 | ENSG00000225611 | lncRNA |
| p3685 | 1.1481086 | 2.902665874 | 3.109362 | 0.008375517 | 0.269394998 | -2.379405 | ENSG00000257700 | lncRNA |
| p37062_v4 | -1.107922 | 3.404109351 | -3.06994 | 0.009033007 | 0.273473567 | -2.445125 | ENSG00000273004 | lncRNA |
| p37068_v4 | -1.16746 | 4.225486936 | -6.14789 | 0.0000364 | 0.11060151 | 2.2071799 | ENSG00000273129 | lncRNA |
| p37093_v4 | 1.0928934 | 4.612622065 | 2.385762 | 0.033109879 | 0.391453277 | -3.566432 | ENSG00000221571 | lncRNA |
| p37116_v4 | 1.5266507 | 7.37401991 | 2.273949 | 0.040742692 | 0.414322461 | -3.742753 | ENSG00000271947 | lncRNA |
| p37140_v4 | 1.0158268 | 4.088865499 | 2.680483 | 0.019007411 | 0.329275105 | -3.090044 | ENSG00000272027 | lncRNA |
| p37277_v4 | -1.009835 | 7.987373297 | -4.55642 | 0.000551243 | 0.187614587 | -0.028898 | ENSG00000226266 | lncRNA |
| p37732_v4 | 1.0282056 | 2.621823294 | 2.165072 | 0.049755517 | 0.43680107 | -3.911419 | ENSG00000271766 | lncRNA |
| p37790_v4 | 1.1973752 | 2.872966083 | 3.808609 | 0.00220544 | 0.226472045 | -1.219594 | ENSG00000230314 | lncRNA |
| p37868_v4 | 1.7031131 | 2.641789899 | 6.520471 | 0.0000203 | 0.097591461 | 2.6659231 | ENSG00000271945 | lncRNA |
| p38179_v4 | -1.083086 | 2.157163288 | -2.66767 | 0.019475343 | 0.329735837 | -3.111031 | ENSG00000226337 | lncRNA |
| p38195_v4 | -1.051021 | 3.837111915 | -2.70187 | 0.018250795 | 0.325171199 | -3.054975 | ENSG00000271314 | lncRNA |
| p3830 | 1.1547389 | 4.375415099 | 4.993248 | 0.000252706 | 0.162553732 | 0.6286424 | ENSG00000257754 | lncRNA |
| p38353_v4 | 1.1487484 | 4.635568312 | 4.800971 | 0.000355141 | 0.174988778 | 0.343071 | ENSG00000271933 | lncRNA |
| p38381_v4 | -1.404998 | 2.550952467 | -2.67928 | 0.019051044 | 0.329404989 | -3.092023 | ENSG00000273143 | lncRNA |
| p38441_v4 | -1.432091 | 2.494249804 | -5.17471 | 0.000184125 | 0.15159608 | 0.892359 | ENSG00000245008 | lncRNA |
| p38522_v4 | 1.0933679 | 4.01520754 | 5.645854 | 0.0000827 | 0.138152128 | 1.550043 | ENSG00000271216 | lncRNA |
| p4078 | 1.0252044 | 3.841180245 | 2.582451 | 0.022886037 | 0.345867886 | -3.250061 | ENSG00000236133 | lncRNA |
| p4185 | 1.5828105 | 2.906006569 | 3.589714 | 0.003340922 | 0.226595231 | -1.57991 | ENSG00000229437 | lncRNA |
| p4191 | 1.4668138 | 3.308039902 | 3.486485 | 0.004067821 | 0.237120028 | -1.751025 | ENSG00000233725 | lncRNA |
| p4225 | -1.010353 | 3.404343507 | -2.62974 | 0.020927524 | 0.335868481 | -3.173038 | ENSG00000236778 | lncRNA |
| p4243 | -1.53739 | 6.582178218 | -3.09078 | 0.008679308 | 0.271207077 | -2.410391 | ENSG00000227611 | lncRNA |
| p4408 | -1.031025 | 2.281188979 | -2.90911 | 0.012291795 | 0.293904475 | -2.712724 | ENSG00000257523 | lncRNA |
| p4554 | -1.080473 | 4.626171658 | -3.48315 | 0.004093822 | 0.237120028 | -1.756566 | ENSG00000258760 | lncRNA |
| p4601 | -1.526367 | 6.72375403 | -2.73202 | 0.017233756 | 0.320791408 | -3.005434 | ENSG00000258819 | lncRNA |
| p4692 | -1.183331 | 4.22715303 | -3.55723 | 0.003554199 | 0.229801266 | -1.633682 | ENSG00000259717 | lncRNA |
| p471 | 1.5564753 | 3.353888299 | 4.297635 | 0.000884766 | 0.203593727 | -0.432554 | ENSG00000227673 | lncRNA |
| p4743 | -1.348909 | 2.806209899 | -3.43053 | 0.004526851 | 0.23960048 | -1.84402 | ENSG00000237054 | lncRNA |
| p4752 | -1.014678 | 2.576715001 | -3.23998 | 0.00651997 | 0.255050829 | -2.161502 | ENSG00000157306 | lncRNA |
| p4764 | 1.1606229 | 3.907450174 | 3.622577 | 0.003138347 | 0.226595231 | -1.525576 | ENSG00000257612 | lncRNA |
| p4770 | 1.4431146 | 3.212390237 | 2.794797 | 0.015291189 | 0.308970928 | -2.901979 | ENSG00000258038 | lncRNA |
| p4832 | 1.5094541 | 2.463828857 | 7.269321 | 0.00000661 | 0.081134697 | 3.5159418 | ENSG00000248550 | lncRNA |
| p4849 | 1.2243292 | 2.964248757 | 3.069894 | 0.009033876 | 0.273473567 | -2.445209 | ENSG00000186369 | lncRNA |
| p4870 | 1.0899945 | 2.9305959 | 2.278064 | 0.04043436 | 0.413631218 | -3.736317 | ENSG00000258422 | lncRNA |
| p4877 | -1.894882 | 3.66059963 | -5.32471 | 0.000142204 | 0.150068671 | 1.1060322 | ENSG00000258517 | lncRNA |
| p4899 | -1.305723 | 5.808225557 | -2.75278 | 0.016565869 | 0.317084071 | -2.971261 | ENSG00000246548 | lncRNA |
| p490 | -1.108621 | 5.616180387 | -2.47868 | 0.027826657 | 0.371757269 | -3.41786 | ENSG00000224515 | lncRNA |
| p491 | -1.177842 | 5.54899939 | -2.8005 | 0.01512588 | 0.308034284 | -2.892568 | ENSG00000231813 | lncRNA |
| p4912 | 1.4476793 | 3.180082564 | 4.578822 | 0.00052932 | 0.185848765 | 0.0055679 | ENSG00000258958 | lncRNA |
| p4929 | -1.285903 | 3.590188196 | -3.00232 | 0.010282957 | 0.282091827 | -2.557776 | ENSG00000259073 | lncRNA |
| p4951 | 1.0370914 | 3.889921488 | 3.442391 | 0.004425346 | 0.2375453 | -1.824292 | ENSG00000258390 | lncRNA |
| p4954 | 1.4158015 | 6.108908047 | 2.828787 | 0.014330946 | 0.305172018 | -2.84581 | ENSG00000187621 | lncRNA |
| p4967 | 1.0932128 | 1.88817235 | 3.290977 | 0.005912962 | 0.249862515 | -2.076456 | ENSG00000258393 | lncRNA |
| p4995 | -1.096724 | 2.925427238 | -2.25937 | 0.041852915 | 0.4183917 | -3.765517 | ENSG00000214548 | lncRNA |
| p5099 | 1.2629988 | 5.05204319 | 3.684829 | 0.002788173 | 0.226472045 | -1.422868 | ENSG00000236914 | lncRNA |
| p5208 | 1.3523408 | 4.121479919 | 2.400456 | 0.032214692 | 0.387075479 | -3.543053 | ENSG00000259316 | lncRNA |
| p529 | 1.2240322 | 3.573967243 | 2.810439 | 0.014841674 | 0.307284955 | -2.876143 | ENSG00000231407 | lncRNA |
| p5373 | 1.248721 | 3.773974912 | 2.810145 | 0.014850009 | 0.307284955 | -2.876629 | ENSG00000259702 | lncRNA |
| p5862 | 1.3191762 | 5.086267852 | 3.464162 | 0.004245008 | 0.237160825 | -1.788105 | ENSG00000261811 | lncRNA |
| p6206 | -1.207831 | 5.168998068 | -5.1825 | 0.000181655 | 0.15159608 | 0.9035609 | ENSG00000261816 | lncRNA |
| p624 | 1.016328 | 3.826235459 | 3.861187 | 0.001997073 | 0.226017267 | -1.133675 | ENSG00000261314 | lncRNA |
| p6286 | -1.171938 | 8.016553487 | -3.15542 | 0.00766752 | 0.263154926 | -2.302575 | ENSG00000260378 | lncRNA |
| p6287 | -1.2041 | 7.978148672 | -3.36813 | 0.00510074 | 0.242854518 | -1.947868 | ENSG00000261158 | lncRNA |
| p6374 | 1.4119597 | 2.690087839 | 3.978985 | 0.001600261 | 0.22540612 | -0.942211 | ENSG00000260958 | lncRNA |
| p6467 | -1.096257 | 2.866594938 | -3.78824 | 0.002292002 | 0.226472045 | -1.252945 | ENSG00000246777 | lncRNA |
| p6498 | -1.322969 | 4.350778775 | -3.87502 | 0.001945684 | 0.226017267 | -1.11112 | ENSG00000260880 | lncRNA |
| p6600 | 1.1376194 | 3.769655046 | 2.20238 | 0.046474442 | 0.428804358 | -3.853989 | ENSG00000180422 | lncRNA |
| p7429 | -1.388532 | 3.276636951 | -3.22612 | 0.006695629 | 0.256602846 | -2.184638 | ENSG00000249870 | lncRNA |
| p7441 | -1.030046 | 5.112295523 | -2.5781 | 0.023074918 | 0.34710565 | -3.25713 | ENSG00000264914 | lncRNA |
| p7449 | -1.041497 | 5.615359202 | -2.74879 | 0.016692164 | 0.318017854 | -2.977829 | ENSG00000267637 | lncRNA |
| p7472 | -1.109557 | 3.543570507 | -3.61947 | 0.003156965 | 0.226595231 | -1.530713 | ENSG00000264754 | lncRNA |
| p7519 | -1.3405 | 7.736666892 | -3.57112 | 0.003461377 | 0.22939667 | -1.610685 | ENSG00000267078 | lncRNA |
| p7781 | -1.033269 | 3.559853693 | -2.28517 | 0.039906857 | 0.412028904 | -3.725188 | ENSG00000267279 | lncRNA |
| p7894 | -1.311354 | 3.858051103 | -3.11785 | 0.008240275 | 0.267967462 | -2.365245 | ENSG00000266805 | lncRNA |
| p7941 | 1.2043493 | 2.32428283 | 3.499629 | 0.003967003 | 0.235144056 | -1.729202 | ENSG00000266237 | lncRNA |
| p7999 | -1.651785 | 3.497339772 | -4.52456 | 0.000584061 | 0.188825047 | -0.078058 | ENSG00000267761 | lncRNA |
| p8094 | 1.8744738 | 2.95202003 | 2.82476 | 0.014441539 | 0.305985693 | -2.85247 | ENSG00000267287 | lncRNA |
| p8238 | -1.890327 | 4.201668587 | -3.87605 | 0.001941892 | 0.226017267 | -1.109432 | ENSG00000268240 | lncRNA |
| p8380 | -1.058318 | 2.798802517 | -2.53779 | 0.024898429 | 0.356992969 | -3.322497 | ENSG00000267044 | lncRNA |
| p8494 | -1.096138 | 2.54176501 | -2.81039 | 0.01484303 | 0.307284955 | -2.876222 | ENSG00000267715 | lncRNA |
| p8547 | -1.083739 | 3.935085221 | -5.24471 | 0.000163148 | 0.151500453 | 0.9925686 | ENSG00000267273 | lncRNA |
| p8550 | -1.288083 | 6.908776688 | -2.51854 | 0.025817606 | 0.360349387 | -3.353615 | ENSG00000267082 | lncRNA |
| p8630 | 1.3485676 | 5.086722205 | 3.057868 | 0.009244536 | 0.27468051 | -2.465251 | ENSG00000268658 | lncRNA |
| p897 | -1.012631 | 3.798862514 | -2.47781 | 0.027872052 | 0.371843228 | -3.419256 | ENSG00000225306 | lncRNA |
| p8990 | 1.2124506 | 2.672257631 | 2.441092 | 0.02985811 | 0.379050888 | -3.478163 | ENSG00000205837 | lncRNA |
| p9127 | 1.1984143 | 2.372584908 | 3.401579 | 0.004784528 | 0.241999872 | -1.892183 | ENSG00000234690 | lncRNA |
| p9163 | -1.504709 | 5.012965392 | -2.87132 | 0.013212597 | 0.299709157 | -2.775386 | ENSG00000238201 | lncRNA |
| p9260 | 1.3991647 | 4.606865084 | 2.813133 | 0.014765597 | 0.307160034 | -2.871692 | ENSG00000239587 | lncRNA |
| p9489 | 1.4902146 | 3.869824115 | 4.424186 | 0.000701326 | 0.194655434 | -0.233926 | ENSG00000231557 | lncRNA |
